# IL-18BP is decreased in osteoporotic women: Prevents Inflammasome mediated IL-18 activation and reduces Th17 differentiation

**DOI:** 10.1038/srep33680

**Published:** 2016-09-21

**Authors:** Mohd Nizam Mansoori, Priyanka Shukla, Manisha Kakaji, Abdul M Tyagi, Kamini Srivastava, Manoj Shukla, Manisha Dixit, Jyoti Kureel, Sushil Gupta, Divya Singh

**Affiliations:** 1 Division of Endocrinology and Centre for Research in Anabolic Skeletal Targets in Health and Illness (ASTHI), CSIR-Central Drug Research Institute, B.S. 10/1, Sector-10, Jankipuram Extension, Lucknow, India; 2 Department of Endocrinology, Sanjay Gandhi Postgraduate Institute of Medical Sciences, Lucknow, India

## Abstract

IL-18BP is a natural antagonist of pro-inflammatory IL-18 cytokine linked to autoimmune disorders like rheumatoid arthritis. However, its role in post menopausal osteoporosis is still unknown. In this study, we investigated the role of IL-18BP on murine osteoblasts, its effect on osteoblasts-CD4+ T cells and osteoblasts-CD11b+ macrophage co-culture. mIL-18BPd enhances osteoblast differentiation and inhibits the activation of NLRP3 inflammasome and caspase-1 which process IL-18 to its active form. Using estrogen deficient mice, we also determined the effect of mIL-18BP on various immune and skeletal parameters. Ovariectomized mice treated with mIL-18BPd exhibited decrease in Th17/Treg ratio and pro-inflammatory cytokines. mIL-18BPd treatment restored trabecular microarchitecture, preserved cortical bone parameters likely attributed to an increased number of bone lining cells and reduced osteoclastogenesis. Importantly, these results were corroborated in female osteoporotic subjects where decreased serum IL-18BP levels and enhanced serum IL-18 levels were observed. Our study forms a strong basis for using humanized IL-18BP towards the treatment of postmenopausal osteoporosis.

Osteoporosis is a disease characterized by low bone strength leading to increased risk of fractures^1^,^2^. It is mainly caused by uncoupling of bone formation and resorption in the bone remodelling^3^,^4^. Bone remodelling is a coordinated process between the formation and degradation of bone, maintained by osteoblasts and osteoclasts and ensures bone homeostasis^5^,^6^. While osteoblasts are the bone forming cells, osteoclasts are the bone resorbing cells. Activation of functional osteoclast requires macrophage colony-stimulating factor (M-CSF) and receptor activator factor of nuclear factor kB ligand (RANKL) secreted by osteoblasts^7^. However, a number of other cytokines and growth factors play important role directly or indirectly in the process of osteoclastogenesis including TNF-α, IFN-γ, IL-17 and IL-1β^8^. Studies have shown that IL-1 receptor antagonist and tumour necrosis factor binding protein decrease osteoclast formation and bone resorption in ovariectomized mice^9^. In fact functional block of pro-inflammatory cytokines like TNF-α and IL-17 prevents bone loss in ovariectomized (Ovx) mice^10^,^11^.

Studies have shown that inflammasome derived IL-1 and IL-18 cytokines promote IL-17 secreting Th17 cell differentiation^12^,^13^. IL-18, also known as IFNγ- inducing factor is an important regulator of both innate and adaptive immune responses, as well as of non-immune mechanisms of host defence and inflammation^14^,^15^. IL-18 is related to IL-1 family, especially IL-1β and similar to it, IL-18 is synthesized as an inactive precursor^15^,^16^. The IL-18 precursor is cleaved by caspase-1 into active IL-18^15^,^16^. Macrophages and dendritic cells are the primary source of active IL-18, whereas the primary precursor remains in the intracellular compartment of mesenchymal stem cells^15^,^16^. IL-18 is a pro-inflammatory cytokine which is implicated in pathophysiology of several autoimmune disorders like inflammatory arthritis^15^,^16^. Recent reports have shown that IL-18 up regulates the production of key osteoclastogenic regulators from fibroblast-like synoviocytes in rheumatoid arthritis (RA)^17^. High levels of IL-18 mRNA and protein have been found in RA synovial tissues. There is a report which states that IL-18-deficient mice evade collagen-induced arthritis (CIA) in a mice RA model^18^. Studies show that the incidence and severity of CIA in mice is worsened by IL-18 administration^19^.

The effects of endogenous IL-18 are regulated by a specific antagonist, the IL-18 binding protein (IL-18BP), a 40-kDa molecule encoded by a separate mRNA that exhibits limited homology to the ligand-binding α-chain of the IL-18R. IL-18BP binds to IL-18 and functions as a competitive inhibitor of receptor binding of IL-18^20^. Studies have shown that murine IL-18 BP treatment reduces the severity of CIA in mice^15^. There are four human and two murine isoforms of IL-18BP which have been identified. The human isoform IL-18BPa and IL-18BPc neutralize IL-18 activity sufficiently at an equimolar ratio while mice IL-18BPc and IL-18BPd isoform neutralize the biological activity of mice IL-18 at an equimolar ratio^21^. IL-18BP is currently in clinical trials for treatment of RA and severe psoriasis^22^,^23^. Though IL-18BP has been used safely in humans and clinical trials of neutralizing anti-IL-18 antibodies as well as IL-18BP are in progress in various diseases^16^, there has been no study on the role of IL-18 in estrogen deficiency induced bone loss. There are reports that patients with endogenous Cushing’s syndrome (CS) who have decreased bone mass and enhanced risk for osteoporotic fractures have raised levels of serum IL-18^24^. Enhanced serum levels of IL-18 are also reported in RA^25^. However, there is no direct study in post menopausal osteoporosis. Moreover, IL-18BP which is an antagonist of IL-18 has never been measured in osteoporotic subjects.

In this study, we investigated whether IL-18 plays a role in pathogenesis of post menopausal osteoporosis and if the exogenous administration of murine IL-18BPd isoform has a protective role in estrogen deficiency induced bone loss. We have determined the (i) effect of mIL-18BP treatment on mice osteoblast cells, (ii) how it affects the T and B cells in estrogen deficient mouse model and finally (iii) how these effects are translated to various skeletal parameters. Importantly, we have measured the levels of serum IL-18BP and serum IL-18 in healthy, osteopenic and osteoporotic post menopausal women to determine if their level co-relate with bone mineral density data.

## Results

### Decreased expression of IL-18BP in mice and human PBMCs

**Figure 1:**
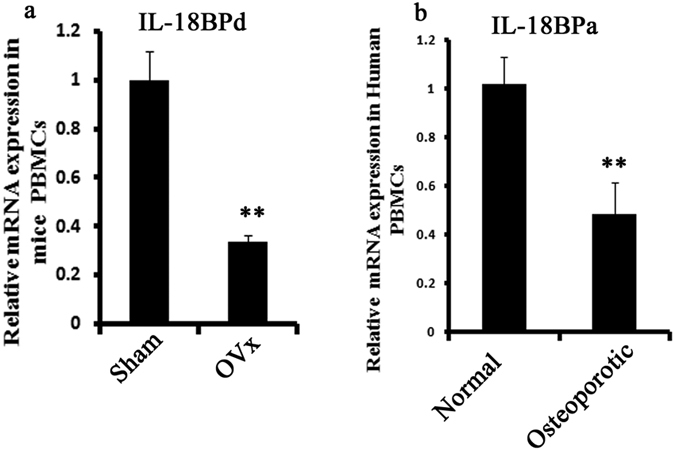
IL-18BPd and IL-18BPa mRNA expression in PBMCs isolated from Ovx mice and osteoporotic human subjects. (**a**) mRNA expression of IL-18BPd were determined in PBMCs of Ovx and Sham animals post one month of Ovx. n = 10 mice/group; data are presented as mean ± SE; **p < 0.01 compared with sham group. (**b**) mRNA expression of IL-18BPa in PBMCs isolated from Normal and Osteoporotic group in Post Menopausal women based on BMD. n = 6 subjects/group; Data represent three independent experiments and expressed as mean ± SE with 95% confidence interval. Student’s t- test was used to compare between two means using Prism version 3.0 software. **P < 0.01 compared with Normal group.

IL-18 plays an important role in the pathophysiology of inflammatory arthritis and the effects of endogenous IL-18 are regulated by IL-18BP^15^. Hence, mRNA transcript levels of IL-18 BPd were evaluated in mouse PBMCs (peripheral blood mononuclear cells) after one month of ovarieoctomy (Ovx). IL-18BP transcript levels were significantly reduced in Ovx animals compared to sham operated (sham) animals (Fig. 1a). Corroborating these data was the expression levels of IL-18BPa observed in PBMCs of osteoporotic patients which was significantly lower than the control subjects (Fig. 1b).

### IL-18BPd administration increases BMP-2 expression in osteoblasts

**Figure 2:**
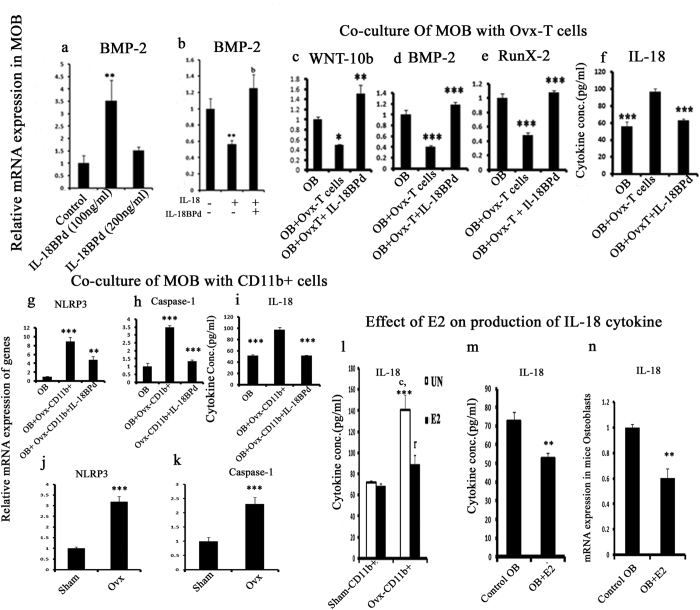
Effect of IL-18BPd on murine osteoblasts alone and in co-culture with CD4+ T cells and CD11b+ cells. (**a**) Calvarial osteoblast cells treated with mIL-18BP (100 ng/ml) for 48h exhibited increased expression of BMP-2. (**b**) Calvarial osteoblasts treated with IL-18 (100 ng/ml) for 48h show reduced BMP-2 expression in contrast to increased expression of BMP-2 when both IL-18 and mIL-18BP are given together. (**c**) Co-culture of CD4+ T cells isolated from Ovx mice and osteoblasts show reduced expression of wnt10b, (**d**) BMP-2, (**e**) Runx-2). This effect is reversed by mIL-18BPd treatment. (**f**) IL-18 ELISA in conditioned media of the co-culture. (**g**) Co-culture of CD11b+ from Ovx mice and osteoblasts show increased expression of NLRP3 inflammasome and (**h**) caspase-1. This effect is reversed by mIL-18BPd treatment. (**i**) IL-18 ELISA in conditioned media of the co-culture. (**j**) Increased NLRP3 and caspase-1 (**k**) gene expression in PBMCs isolated from Ovx mice compared to sham. (**l**) IL-18 ELISA in conditioned media of CD11b+ macrophages harvested from Ovx and sham group mice and treated with E2 (**m**) IL-18 ELISA in conditioned media of osteoblast cells treated with or without E2. (**n**) IL-18 mRNA expression in osteoblast cells treated with or without E2. Data represent three independent experiments and expressed as mean ± SE with 95% confidence interval. Statistical analysis was performed by ANOVA method followed by the Newman–Keuls test of significance using Prism version 3.0 software. **p < 0.01 compared with sham (control OB group); bp < 0.01 compared between IL-18 vs IL-18 + mIL-18BP treatments. ***p < 0.001 compared with sham-CD11b + group; ^c^p < 0.001 compared with sham CD11b + treated with E2 group. ^r^p < 0.001 compared with Ovx-CD11b + .

As IL-18 promotes IL17 production, which inhibits osteoblast differentiation and mineralization, we assessed the effect of IL-18 and IL-18BP on BMP-2 transcript level which is an important osteoblast differentiation marker. To determine the effect of exogenous mIL-18BP treatment on osteoblasts, cells were treated with mIL-18BP at concentrations of 100 ng/ml and 200 ng/ml for 48 h. Cells were harvested for RNA isolation and gene expression levels of BMP-2 were determined. It was observed that mIL-18BP treatment at 100 ng/ml significantly elevated the BMP-2 mRNA transcript levels (Fig. 2a). In contrast, in osteoblast cells treated with IL-18 at 100 ng/ml, gene expression level of BMP-2 was significantly down regulated. However, the expression level of BMP-2 was restored back to control levels when cells were treated with IL-18 BP along with IL- 18 (Fig. 2b).

### IL-18BPd inhibits the effect of Ovx-CD4+ T cells and Ovx-CD11b+ macrophages on osteoblasts and decreases IL-18 production

PBMCs isolated from Ovx mice exhibited low IL-18BP mRNA levels. Thus, it was decided to study the effect of IL-18BPd on osteoblasts co-culture with CD4+ T cells and CD11b+ macrophages isolated from Ovx mice. It was observed that expression levels of WNT-10b, Runx-2 and BMP-2 were decreased in osteoblast cells co-cultured with CD4+ T cells isolated from Ovx mice. However, treatment of IL-18BPd to the co-culture resulted in increased mRNA expression of the WNT-10b, BMP-2 and Runx-2, thus abolishing the inhibitory effect of CD4+ T cells isolated from Ovx mice (Fig. 2c–e). IL-18BPd treatment to the co-culture also led to decreased IL-18 production in the conditioned media, which was otherwise increased in osteoblasts incubated with CD4+ T cells derived from Ovx mice (Fig. 2f).

Co-culture of mice osteoblasts with Ovx-CD11b+ cells also enhanced the level of NLRP3 inflammasome and Caspase-1 which are involved in IL-18 activation (Fig. 2g,h). However, treatment with IL-18BPd abolished this effect (Fig. 2g,h). As NLRP3 and caspase-1 mediate the processing of precursor IL-18 to its mature form, IL-18 production was assessed in these co-cultures. IL-18BPd treatment to the co-culture also led to decreased IL-18 production in the conditioned media which was otherwise increased in osteoblasts incubated with CD11b+ macrophages derived from Ovx mice (Fig. 2i). Transcript levels of NLRP3 and caspase-1 were also determined in PBMCs harvested from Ovx and sham animals. Increased expression of NLRP3 and caspase-1 were found in Ovx animals compared to sham group (Fig. 2j,k). Expression of cleaved caspase-1 was also analyzed by FACS in macrophages isolated from sham and Ovx groups. Caspase-1 expression was significantly enhanced in Ovx macrophages while IL-18BPd treatment led to significant decrease in caspase-1 expression in Ovx macrophages (Supplementary Fig. 2a,b). Sham macrophages treated with mL-18BPd has no significant difference in caspase-1 expression when compared with sham untreated group (Supplementary Fig. 2a,b).

IL-18 production was also determined in CD11b+ macrophages derived from the sham and Ovx mice in the presence or absence of estrogen. In CD11b+ macrophages derived from Ovx mice, IL-18 levels were significantly higher. However treatment with estrogen led to significant down regulation in IL-18 levels (Fig. 2l). A similar effect was seen in osteoblast cells where estrogen treatment to osteoblasts led to decrease in IL-18 production in the conditioned media (Fig. 2m). IL-18 gene expression was also determined in osteoblast cells. Treatment with estrogen led to significant decrease in IL-18 mRNA expression compared to untreated osteoblast cells (Fig. 2n). Effect of estrogen on IL-18BPd gene expression was determined in mouse osteoblasts and it was observed that treatment with estrogen has increased IL-18BPd expression in mouse osteoblasts when compared with control group (Supplementary Fig. S4b).

### IL-18BPd inhibits immunosenecence and B-lymphopoesis in Ovx mice

**Figure 3:**
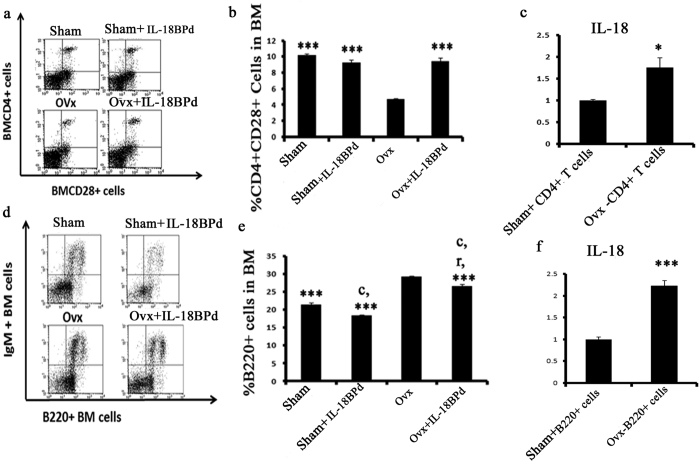
Effect of mIL-18BP on Ovx‐induced increases in CD4+CD28+ T cells and B cells. (**a,b**) The CD4+CD28+ T‐cell population was decreased in the BM of Ovx mice and mIL-18BP treated group reversed this loss of CD28. No difference was observed in sham and sham group treated with mIL-18BPd (**c**) IL-18 transcript levels in CD4+ T cells isolated from Ovx and sham group mice. (**d,e**) The population of B220+ cells was increased in BM of Ovx mice and mIL-18BP significantly decreased the percentage of B220+ cells. (**f**) IL-18 transcript levels in B220+ cells isolated from Ovx and sham group mice. Statistical analysis was performed by ANOVA method followed by the Newman–Keuls test of significance using Prism version 3.0 software. n = 10 mice/group; data are presented as mean ± SE; ***p < 0.001 compared with Ovx; ^c^p < 0.001 compared with Sham group; ^r^p < 0.001 compared with Sham + mIL-18BPd. Data represent three independent experiments and expressed as mean ± SE with 95% confidence interval. Two tailed student’s t- test was used to compare between two means using Prism version 3.0 software.*P < 0.05 compared with sham-CD4+ group.**P < 0.01 compared with sham- B220+ group.

Above results demonstrated that co-culture of CD11b+ cells isolated from Ovx mice with mice osteoblasts exhibited an enhanced level of NLRP3 inflammasome. NLRP3 inflammasome is known to promote immunosenescence^26^. Also, studies in our lab have shown that Ovx leads to premature T cell senescence due to loss of CD28 expression, an immunosenescence marker^27^. Hence, the effect of exogenous mIL-18BP treatment was studied on Ovx induced CD28 loss on CD4+ T cells. Prior to that, serum levels of IL-18 were determined in various groups in order to confirm that exogenous supplementation of mIL-18BPd satisfactorily neutralized circulatory IL-18. Ovx induced high serum IL-18 levels were significantly reduced in mIL-18BPd treated Ovx mice (Supplementary Fig. S1). Sham treated with mIL-18BPd also exhibited significant reduction of IL-18 when compared with the sham group (Supplementary Fig. S1). On evaluation of CD4+CD28+ T cell population in various groups, it was observed that CD4+CD28+ T cell population in BM of the Ovx group was dramatically reduced compared with the sham group. CD4+CD28+ T cell population in BM of the sham and sham + mIL-18BP groups were not different (Fig. 3a,b). CD4+CD28+ T cell population in BM of the sham and Ovx+mIL-18BP was also comparable (Fig. 3a,b). In order to see if increased IL-18 levels are responsible for less CD4+CD28+ cells in Ovx mice, IL-18 mRNA levels were determined in CD4+ T cells isolated from Ovx and sham group. It was observed that IL-18 mRNA levels were significantly higher in CD4+ T cells isolated from Ovx mice compared to sham group (Fig. 3c).

There is also a report where NLRP3 inflammasome has been shown to be critical for B cell activation^28^. Besides, Ovx is known to increase the proliferation of B220+ cells in BM while estrogen treatment completely reverses this change^29^. Hence, effect of mIL-18BPd was also studied on B220+ cell proliferation. Mice Ovx for 4 weeks had a higher number of B220+ cells in BM compared with the sham group. Treatment of Ovx mice with mIL-18BP diminished the Ovx-induced increase in B220+ population. (Fig. 3d,e). Interestingly; sham group treated with IL-18BPd resulted in a decrease in no. of B220+ cells (Fig. 3d,e). IL-18 mRNA levels were also determined in B220+ cells isolated from the sham and Ovx mice and increased expression of IL-18 was observed in B220+ cells harvested from Ovx mice (Fig. 3f).

### Effect of mIL-18BP on Th17/Treg balance

**Figure 4:**
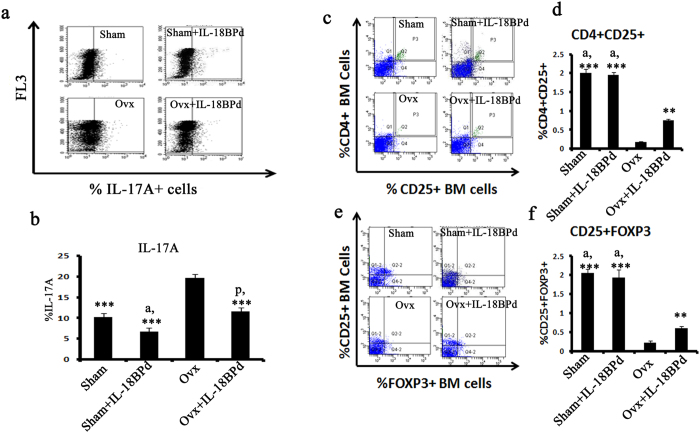
Effect of mIL-18BP on Th17/Treg balance studied using PBMCs. (**a**) Ovx induces the proliferation of Th17 cells while administration of mIL-18BP reduces the number of Th-17 cells. (**b**) Quantification of the FACS data. Percentage of Th17 cells was higher in Ovx animals and treatment with mIL-18BPd inhibited this increase. (**c,d**) Percentage of CD4+CD25+ cells and (**e,f**) CD25+FOXP3+ cells was measured by FACS. Percentage of CD4+CD25+ cells and CD25+FoxP3+ cells were significantly lower in Ovx group animals. Treatment of Ovx mice with mIL-18BP led to significant increase in CD4+CD25+ and CD25+FoxP3+ Treg cells. n = 10 mice/group; data are presented as mean ± SE; ***p < 0.001 compared with Ovx group; **p < 0.01 compared with Ovx group; ^a^p < 0.05 compared with Ovx+mIL-18BPd group.

IL-18 is cleaved into its activated form by caspase-1 and inflammasome which drives Th17 responses. Hence the percentage of Th17 cells were determined in sham, sham + IL-18BPd, Ovx and Ovx+ IL-18BPd groups. Ovx leads to an up regulation of IL-17 secreting Th17 cells^10^. As expected, the Ovx group had higher population of IL-17A^+^ cells (P < 0.01) in the Spleenocytes compared to the sham (Fig. 4a,b). mIL-18BP treatment to Ovx mice presented with decreased IL-17^+^ cells (P < 0.01) and was comparable to sham group (Fig. 4a,b). No difference was observed between sham and sham treated with mIL-18BPd groups.

There exist a molecular antagonism between Th17 and T regulatory cells^30^, hence percentage of CD4+CD25+ FoxP3 cells was also determined in various groups. For determining the percentage of CD4+CD25+ and CD25+FoxP3+, PBMCs were isolated and intracellular staining of FoxP3 was carried out. Percentage of CD4+CD25+ cells and CD25+FOXP3+ cells was measured by FACS. The results showed that percentage of CD4+CD25+ cells were significantly lower in Ovx group animals compared to sham group animals (P < 0.001). Treatment of Ovx mice with mIL-18BP led to significant increase in CD4+CD25+ Treg cells (P < 0.001), albeit less than the sham group (Fig. 4c,d). FoxP3 is the major transcription factor regulating Treg cell development^10^,^30^. On examining the FoxP3 expression on CD4+CD25+ cells, a significant decrease was observed in Ovx animals compared to sham (P < 0.001). mIL-18 BP treatment of Ovx animals reverted the loss in Foxp3 expression (P < 0.01) though significantly less than the sham group (Fig. 4e,f). FoxP3 expression between sham and sham + mIL-18BPd group was not changed.

### Effect of IL-18BPd on transcription factors regulating Th17 and Treg development

**Figure 5:**
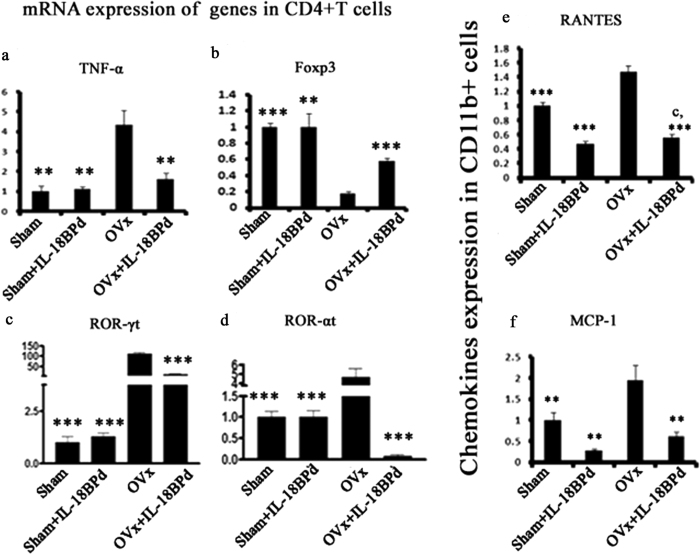
Relative mRNA expression of (**a**) TNF-α, (**b**) FoxP3, (**c**) ROR-α, and (**d**) ROR-γt, in isolated CD4^+^ cells was measured in all the groups. Relative mRNA expression of (**e**) RANTES and (**f**) MCP-1 in isolated CD11b+ cells was measured from all the groups. Data represent three independent experiments and expressed as mean ± SE with 95% confidence interval. Statistical analysis was performed by ANOVA method followed by the Newman–Keuls test of significance using Prism version 3.0 software. ***p < 0.001 compared with Ovx group; **p < 0.01 compared with Ovx + sham; *p < 0.05 compared with Ovx group.

Transcription factors like RORγt and RORα play a vital role in Th17 cell development^31^. Likewise, Foxp3 is important for Treg cell differentiation^31^. Hence, effect of mIL-18BP treatment was determined on the transcription factors governing Th17 and Treg development. It was observed that while FoxP3 mRNA levels in CD4+ T cells were significantly lower in Ovx mice (P < 0.001), RORγt (P < 0.001) and RORα (P < 0.01) (transcription factors controlling Th17 development)^10^,^30^ were significantly higher in Ovx animals compared to sham (Fig. 5b–d). However, exogenous supplementation of mIL-18BP to Ovx mice led to increased FoxP3 transcript levels (P < 0.001) and decrease in RORα (P < 0.01) and RORγt (P < 0.001) mRNA levels (Fig. 5b–d). Additionally, pro-inflammatory osteoclastogenic cytokines like TNF-α (P < 0.01) was also elevated in Ovx mice compared to sham but treatment with mIL-18BP reduced the levels of these cytokines in Ovx animals (Fig. 5a). No difference was observed between sham and sham group treated with mIL-18BPd.

### Effect of IL-18BPd on CD11b+ Macrophages and pro-inflammatory/anti-inflammatory cytokine production

IL-18 is one of the most important innate cytokines produced from macrophages in the early stages of the inflammatory immune response^32^. IL-18 is also known to induce the production of chemokines like MCP-1 (Monocyte chemoattractant protein-1) and RANTES^32^. Production of MCP-1 and RANTES from CD11b+ macrophages was assessed in estrogen deficient conditions. In Ovx mice levels of RANTES and MCP-1 were significantly higher compared to sham group animals (P < 0.001 and P < 0.01, respectively) (Fig. 5e,f). However, supplementation of mIL-18BP to Ovx animals mitigated this effect (Fig. 5e,f). Interestingly, transcript levels of RANTES and MCP-1 were significantly reduced in sham group treated with mIL-18BPd and were even comparable to Ovx + mIL-18BPd treated groups.

Aberrant immune response contributes to the pathogenesis of postmenopausal osteoporosis via an altered pro inflammatory-to-anti inflammatory cytokine balance^33^. Therefore, we examined the effect of mIL-18BP treatment on the levels of major pro-inflammatory and anti-inflammatory cytokines. In the Ovx group, there was a significant increase in the levels of IL-2, IL-6, IL-17, IFN-γ and TNFα and decrease in IL-10 over the sham group (Supplementary Table 1). However, treatment of Ovx animals with mIL-18BP significantly reduced the levels of these pro-inflammatory cytokines and brought it back to sham level and even better than sham in case of TNF-α and IFN-γ (Supplementary Table 1). The levels of pro-inflammatory cytokines were not different between sham and sham + mIL-18BPd except TNF-α and IFN-γ which were significantly reduced in sham + mIL-18BPd (Supplementary Table 1).

### Effect of exogenous mIL-18BP supplementation on trabecular microarchitecture of Ovx mice

**Figure 6:**
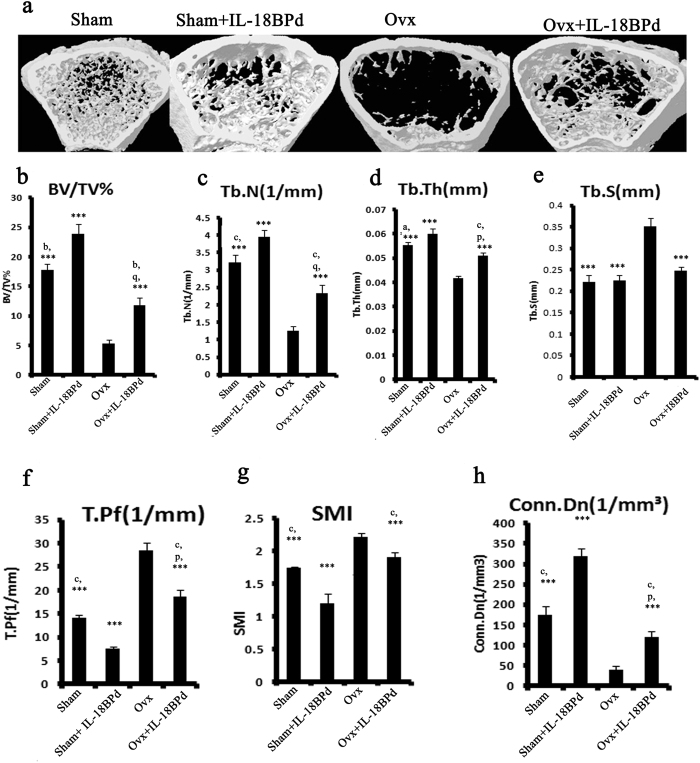
Effect of mIL-18BP on Ovx induced bone loss and deterioration of trabecular microarchitecture in femur bone. (**a**) Representative images of femoral bones of different groups (**b**) BV/TV, (**c**) Tb.N., (**d**) Tb.Th, (**e**) Tb.Sp, (f) Tb.Pf, (**g**) SMI and (**h**) Conn. D. Administration of mIL-18BP restores Ovx induced alterations in femur trabecular region. Statistical analysis was performed by ANOVA method followed by the Newman–Keuls test of significance using Prism version 3.0 software. n = 10 mice/group; data are presented as mean ± SE; ***p < 0.001 compared with Ovx group; **p < 0.01 compared with Ovx group; *p < 0.05 compared with Ovx group; ^c^p < 0.001 compared with sham+mIL-18BPd group; ^b^p < 0.01 compared with sham + mIL-18BPd; ^a^p < 0.05 compared with sham+mIL-18BPd group; ^p^P < 0.05 compared with sham and; ^q^P < 0.01 compared with sham.

In gross observation by 3D-μCT, deterioration of the micro architecture due to destruction of trabeculae of femur bone was readily observed in Ovx group compared with sham group (Fig. 6). Femoral response to various treatments was quantified. Representative 3D image of trabecular bone shows Ovx induced loss of trabecular bone and treatment of IL-18BPd prevented this loss (Fig. 6a). The Ovx group had reduced trabecular bone volume (BV/TV) (P < 0.001) (Fig. 6b), trabecular number (Tb.N) (P < 0.001) (Fig. 6c) and trabecular thickness (Tb.Th) (P < 0.001) (Fig. 6d); and increased trabecular separation (Tb.Sp) (P < 0.001) (Fig. 6e), trabecular pattern factor (Tb.Pf) (P < 0.001) (Fig. 6f) and structure model index (SMI) (P < 0.001) (Fig. 6g) compared to sham group. Ovx also led to decreased connective density (conn. Dn) (P < 0.001) (Fig. 6h) compared to sham group. Treatment of Ovx animals with mIL-18BP led to significant restoration of trabecular parameters as evident by increased BV/TV (P < 0.001) (Fig. 6b), Tb.N (P < 0.001) (Fig. 6c) and Tb.Th (P < 0.001) (Fig. 6d); and decreased Tb.Sp (P < 0.001) (Fig. 6e), Tb.Pf (P < 0.001) (Fig. 6f) and SMI (P < 0.01) (Fig. 6g). mIL-18BP treatment also led to increased Conn. Dn. Compared to Ovx control (P < 0.01) (Fig. 6h). Interestingly, treatment of mIL18BPd to sham group also led to significant enhancement in BV/TV (P < 0.01), Tb.N. (P < 0.001), Tb.Th (P < 0.001), Conn. Density (P < 0.001) and a reduction in SMI (P < 0.001) and Tb.Pf (P < 0.001) compared to sham group. (Fig. 6b–d,f–h). Similar results were obtained in trabeculae of tibia bone of different groups (Supplementary Fig. S6).

### Effect of exogenous mIL-18BP supplementation on cortical bone, osteoblasts and osteoclasts functions

**Figure 7:**
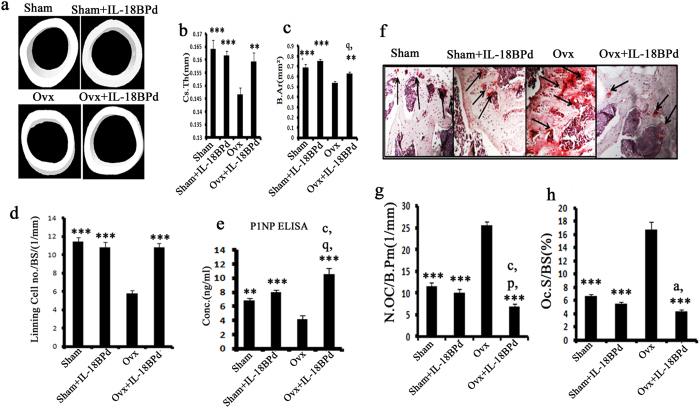
Effect of mIL-18BPd on cortical bone parameters, osteoblast and osteoclast functions. (**a**) Representative 3D microCT images of cortical bone at the femoral diaphysis. (**b**) Measurement of Cs.Th and (**c**) B.Ar. Cortical parameters were decreased in Ovx and mIL-18BP administration to Ovx animals abolished these effects. (**d**) mIL-18BPd treatment to Ovx animals maximally stimulated the production of lining cells on femoral surface compared to control Ovx group and was equivalent to sham and sham treated groups. (**e**) mIL-18BP reversed Ovx induced decrease in serum P1NP and this effect was better than sham and sham treated with mIL-18BPd. Histomorphometric analysis of femoral epiphysis (**f**) TRAP staining (**g**) increased number of osteoclasts (OC)/bone perimeter (N.Oc/B.Pm)and (**h**) osteoclast surface/bone surface (%) in the Ovx group over sham. mIL-18BPd treatment significantly reversed the Ovx-induced changes on osteoclasts. n = 10 mice/group; data are presented as mean ± SE; ***p < 0.001 compared with Ovx group; **p < 0.01 compared with Ovx group; *p < 0.05 compared with Ovx; ^a^p < 0.05 compared with sham group; ^p^p < 0.05 compared with sham + mIL-18BPd; ^q^p < 0.01 compared with sham + mIL-18BPd and ^r^p < 0.001 compared with sham + mIL-18BPd.

2D-μCT measurements at the site of femur mid-diaphysis showed that relative to sham group, Ovx group had decreased cortical thickness (Cs.Th) (P < 0.01) and cortical bone area (B.Ar) (P < 0.01) (Fig. 7a–c). Compared to Ovx group, mIL-18BP treated group showed greater Cs.Th (P < 0.01) and B. Ar (P < 0.05) (Fig. 7a–c). Cortical thickness and cortical bone area were not different between sham and sham group treated with mIL-18BPd (Fig. 7a–c).

In the 3-point bending test of femur, Ovx group exhibited decreased stiffness (P < 0.001) (Supplementary Fig. S6a), energy (P < 0.001) (Supplementary Fig. S6b) and power (P < 0.001) (Supplementary Fig. S6c) parameters compared to sham group. mIL-18BP treatment to Ovx mice led to increased stiffness (P < 0.01), power (P < 0.01) and energy (P < 0.05) compared to Ovx control group (Supplementary Fig. S6a–c). No difference was observed in sham and sham group treated with mIL-18BPd.

As reactivation of quiescent osteoblasts constitutes a major osteogenic response^34^ we quantified lining cell number in various groups. Lining cells were significantly reduced in Ovx mice compared to sham group (P < 0.001). Treatment of Ovx mice with mIL-18BP led to significant increase in number of lining cells (P < 0.001) (Fig. 7d). Number of lining cells was not different between sham and sham + mIL-18BPd treated groups. Also, serum P1NP (total procollagen type 1 N-terminal propeptide) levels, an accepted osteogenic marker^35^, were significantly reduced in Ovx mice compared to the sham (Fig. 7e). P1NP levels were significantly increased in mIL-18BP treated Ovx animals; in fact the increase observed was more than the sham and sham group treated with mIL-18BPd (Fig. 7e).

Histomorphometric analysis of decalcified femurs stained with TRAP (Tartarate resistant acid phosphatase), an osteoclast marker, showed increased TRAP staining in Ovx mice which was indicative of enhanced osteoclast numbers compared to sham group. However, in Ovx animals treated with mIL-18BP, reduced TRAP staining was observed suggesting decreased osteoclast activity (Fig. 7f). Osteoclast number and osteoclast surface area was increased in Ovx group while treatment with mIL-18BPd reduced osteoclast no. and osteoclast surface area (P < 0.001) (Fig. 7g,h). The decrease observed was in fact more than that observed with sham and sham treated with mIL-18BPd (Fig. 7g,h).

Apart from IL-18, IL-18 BP is also known to bind to IL-37 cytokine in humans^36^. Though the mouse isoform of IL-37 has not been discovered^36^, it was still ascertained that effects observed on skeletal and immune parameters were due to inhibition of IL-18 by IL-18BP and not due to binding of IL-37 to IL-18BP. For this sham and Ovx mice were treated with IL-18 neutralizing antibody. Following this, immune parameters like percentage of TH17 and Treg cells and osteogenic gene expression in bone tissue and osteoblast-Ovx T cell co-culture were determined. It was observed that percentage of IL-17A+ve cells were significantly higher in Ovx animals, however, treatment with neutralizing antibody against IL-18 reduced the percentage of IL-17+ve cells (Supplementary Fig. S5a,b). On the contrary, FoxP3+ve cells were lower in Ovx animals while its percentage significantly increased in Ovx animals treated with neutralizing IL-18 antibody (Supplementary Fig. S5c,d). Bone tissue isolated from Ovx animals exhibited low transcript levels of Runx-2 and BMP-2, however, treatment of Ovx animals with neutralizing IL-18 antibody up regulated Runx-2 and BMP-2 mRNA expression (Supplementary Fig. S5e,f). Similarly gene expression of Runx-2 and BMP2 in co-cultures of osteoblasts and T cells isolated from Ovx mice was significantly low. On the contrary, osteoblasts co-cultured with T cells isolated from Ovx animals and treated with IL-18 neutralizing antibody presented with increased expression of Runx-2 and BMP-2 (Supplementary Fig. S5g,h). Thus, these experiments prove that effects observed by treatment of Ovx animals with IL-18BP are due to its binding and inhibition of IL-18 cytokine.

### Serum level of IL-18, IL-18BPa in osteoporotic patients and correlation of IL-18BPa with BMD in Post-menopausal women

**Figure 8:**
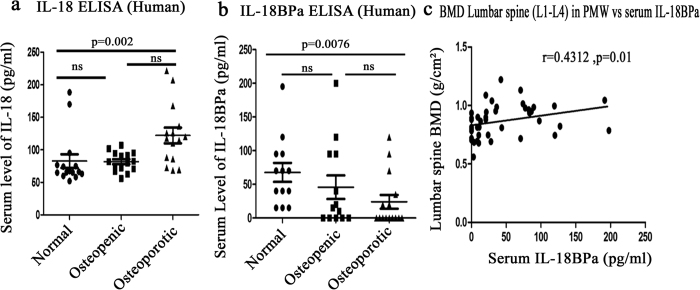
Levels of hIL-18, hIL-18BPa in healthy, osteopenic and osteoporotic subjects and its correlation with BMD data. (**a**) hIL-18 levels in normal, osteopenic and osteoporotic post menopausal women. (**b**) hIL-18BPa levels in normal, osteopenic and osteoporotic post menopausal women. (**c**) Correlation with hIL-18BPa serum levels and BMD values. Kruskal-Wallis test was used for statistical analysis followed by Dunn’s Multiple comparison test. Data analysis was done using Graph Pad prism version 5.0 software. Correlation between variables was performed using Pearsons’ correlation test. Values of p < 0.05 and were considered significant. (**a**) Comparison between normal and osteoporotic group shows p = 0.002. (**b**) Comparison between normal and osteoporotic group shows p = 0.0076. Correlation test between BMD and serum level of IL-18BPa in post menopausal women shows r = 0.4312 and P = 0.01.

Raised levels of serum IL-18 are found in patients of RA and Cushing syndrome which have decreased bone mass and increased risk of osteoporotic fractures^24^. Hence, it was deemed reasonable to detect the levels of IL-18BP which is an IL-18 antagonist in osteoporotic subjects. This would further validate our observations obtained in the mice model. Moreover, as per our knowledge, serum IL-18BP levels have never been measured in human osteoporotic subjects. For this study, human blood was obtained from 45 post menopausal women. Bone mineral density (BMD) of post –menopausal women was analyzed using DXA and based on T-score value they were divided in three groups viz. normal, osteopenic and osteoporotic with 15 subjects in each group. The mean age was 63.86 ± 2.68, 61.46 ± 1.99 and 64.23 ± 1.23 years of normal, osteopenic and osteoporotic subjects respectively. Serum levels of IL-18 and IL-18BPa were measured in all the three groups. It was found that serum level of IL-18 was significantly increased in osteoporotic group compared to the normal group (Fig. 8a). On the contrary, IL-18BPa was decreased significantly in osteoporotic group in comparison with normal group (Fig. 8b). Also, serum levels of IL-18BPa in 45 post menopausal women showed a positive correlation with BMD (Fig. 8c). Thus high serum IL-18BP levels are associated with lower risk of osteoporosis in postmenopausal women.

## Discussion

IL18 is a cytokine that belongs to the IL-1 super family and is mainly produced by macrophages and dendritic cells^32^. IL-18 is able to induce severe inflammatory reactions and plays role in the pathogenesis of various inflammatory disorders like CIA^15^ and acute and chronic DSS-induced colitis^37^. There are indirect studies which suggest that IL-18 levels are increased in osteoporosis, for instance in Cushing’s syndrome (CS) where patients have decreased bone mass and enhanced risk for osteoporotic fractures, high IL18 serum levels are found to be prevalent^24^. However, there are no direct studies on the role of IL-18 in pathogenesis of post menopausal osteoporosis and if inhibiting this inflammatory cytokine may lead to any osteoprotective effects. IL-18BP is a naturally occurring protein that binds to and neutralizes IL-18^20^. IL-18BP have protective role in various diseases like CIA^15^, acute and chronic DSS-induced colitis^37^ and atherosclerosis in apolipoprotein E–deficient mice^38^. However, its role has not been studied in post menopausal osteoporosis. In this study, we report for the first time the immunoprotective and osteoprotective effects of mIL-18BP administration in estrogen deficient Ovx mouse model. We have also analyzed the serum level of hIL-18BP and IL-18 in human subjects to validate our hypothesis. Furthermore, serum level of hIL-18BP shows positive correlation with BMD data of post menopausal women.

To validate our target we first analyzed mRNA expression level of mIL-18BP in PBMCs isolated from one month old Ovx mice. We found that expression level of mIL-18BP was decreased in comparison with sham group. We also found decreased expression level of hIL-18BPa in PBMCs isolated from osteoporotic subjects when compared with normal post menopausal subjects. Previous studies by our group have shown that estrogen deficiency enhanced Th17 differentiation which produces IL-17 cytokine which in turn has an inhibitory effect on osteoblasts differentiation and mineralization^10^. Thus, we decided to study the role of IL-18 and its binding protein IL-18BP on murine osteoblasts. It was found that IL-18BP treatment to osteoblast cells led to enhanced transcript levels of BMP-2, an important osteoblast differentiation marker. On the other hand, IL-18 treatment led to a reduction in BMP-2 mRNA expression, an effect that was abolished on co-treatment of mIL-18BP. Thus, IL-18 exerts a negative effect on osteoblast differentiation. Our results are in contradiction with reports where IL-18 is shown to up regulate OPG production in osteoblast cells^39^ and also mediate a major part of Endothelin-1 induced osteoblasts proliferation^40^. Additionally reports are there which have shown that IL-18 acts via GM-CSF to inhibit osteoclast formation^41^. However, there is also a study by Dai *et al*. who have shown that IL18 stimulates osteoclast formation through synovial cells in RA^42^.

Further, in order to understand the mechanism through which IL-18BP exerts positive effect on osteoblast differentiation, co-culture of osteoblasts with CD4+ T cells isolated from Ovx mice was carried out. In this case low expression of Wnt10b, Runx-2 and BMP-2 which enhance osteoblast differentiation was observed. However, this effect was reversed by pre-treatment of co-cultures with IL-18BP. Co-culture of osteoblasts with CD4+ T cells isolated from Ovx mice also enhanced IL-18 production from osteoblasts. This increase was mitigated on addition of IL-18BP in the co-cultures. Additionally, co-culture with CD11b+ macrophages isolated from Ovx mice also leads to a surge in IL-18 production from osteoblasts, an effect that was mitigated when IL-18BP was added in the co-cultures. Treatment of IL-18BP to the co-cultures antagonizes IL-18 and thus protects the osteoblasts from its detrimental effects.

There are reports that constitutively activated inflammasome leads to abnormal skeletal development in mice^43^. NLRP3 inflammasome is expressed by osteoblasts and in fact treatment with LPS or TNF-α increases its expression^43^. NLRP3 inflammasome and caspase-1 are involved in activation of cytokines like IL-1β and IL-18^43^. It was observed that expression of NLRP3 and caspase-1 was higher in co-cultures of osteoblasts with CD11b+ cells isolated from Ovx mice. However, pre-treatment of co-cultures with IL-18BP led to significant reduction in NLRP3 and caspase-1 expression. This data was also corroborated by increased NLRP-3 and caspase-1 gene expression in PBMCs harvested from Ovx animals compared to sham. Protein expression of cleaved caspase-1 was also analyzed in macrophages isolated from sham and Ovx mice groups. Macrophages isolated from Ovx groups have shown an increased expression of caspase-1 while treatment with IL-18BPd has siginificantly reduced this effect. Thus, we speculate that in Ovx conditions inflammasome activation stimulates caspase-1 to process precursor IL-18 to its mature form thus causing deleterious effects on bone. IL-18BP might inhibit this phenomenon by preventing inflammasome mediated IL-18 activation. Altogether, these results support the protective effect of IL-18BP on osteoblasts and its inhibitory role in inflammasome mediated activation of IL-18 cytokine.

In order to study the effect of estrogen on IL-18 levels, CD11b+ macrophages from Ovx and sham mice or osteoblasts were treated with estrogen and IL-18 production was estimated. We observed that estrogen treated macrophages and osteoblasts produced significantly less levels of IL-18 compared to untreated cells. Estrogen treatment also reduced IL-18 mRNA expression in osteoblast cells. Thus, E2 definitely down regulates IL-18 production. Further, to see if IL-18 expression is altered in other tissues as well in Ovx conditions, its transcript levels were checked in uterine tissue from Ovx and sham mice. Expression of IL-18 was found to be enhanced in uterine tissue of Ovx mice compared to sham (Supplementary Fig. S4). In fact, a study by Murakami *et al*.^44^ has shown that estrogen inhibits IL-18 production in the mouse uterus. Also another report by Xu *et al*.^45^ has shown that ovariectomy increased the levels of cytokines like IL-1β and IL-18, NLRP3 expression and active caspase-1 in hippocampus of female mice. Estrogen treatment has also increased mRNA expression of IL-18BPd in mouse osteoblasts compared with control group.

Our data suggested that IL-18BP prevents inflammasome activation and there are studies which propose that NLRP3 inflammasome are involved in age relate immunosenescence and B cell activation^26^,^28^. Thus, we decided to study the impact of exogenous supplementation of IL-18BP on T cell senescence, which increases the production of pro-inflammatory cytokines and B cells which are an important source of RANKL, the main osteoclastogenic cytokine. Ovx animals and sham groups were treated with or without mIL-18BPd and CD4+ T cells were isolated. We evaluated the loss of CD28, the major marker of immunosenescence on these T cells. It was observed that T cells from Ovx mice presented with increased number of CD28 null cells while treatment with IL-18BPd reversed this effect. Senescent T cells produce large amounts of TNF-α. One of the TNF-α inducing factor is IL-18^46^. Hence mRNA transcript levels of IL-18 were checked in CD4+ T cells from Ovx and sham mice. Increased IL-18 expression was found in CD4+ T cells from Ovx mice. Thus, IL-18 may be increasing TNF production from senescent T cells in estrogen deficient conditions. In case of B cells harvested from Ovx mice also increased transcript levels of IL-18 were found. However, treatment with mIL-18BPd led to reduction in Ovx induced increase in B cells. Interestingly, sham treated with mIL-18BPd showed significant reduction in B cells compared to sham.

The next important aspect was to study the role of IL-18BP on TH17/Treg ratio. Ovx leads to an increase in TH17 cells and a decrease in Treg cells. NLRP3 inflammasome and caspase-1 which produce an active form of IL-18 drive TH17 responses^47^. IL-18 itself promotes Th17 production. We observed that TH17 producing IL-17 cells were significantly higher in Ovx mice while mIL-18BPd treatment inhibited this effect. Again, sham treated with mIL-18BPd presented with reduced percentage of IL17A+ cells compared to sham. This suggested the immunoprotective potential of IL-18BP in normal conditions also. Contrary to IL-17, FoxP3+ cells were reduced post ovx; however, mIL-18BPd significantly restored the FoxP3 positive cell population. The effect though was not as robust compared to sham. These observations suggest that IL-18BP primarily acts by inhibiting IL-18 mediated Th17 production. In agreement with these observations, transcription factors governing TH17 differentiation were up regulated in CD4+ T cells isolated from Ovx mice while mIL-18BPd treatment diminished these effects. mIL-18BPd treatment also reduced the expression levels of chemokines like RANTES and MCP-1 from CD11b+ cells whose production is driven by IL-18. Additionally, mIL-18BPd treatment to Ovx mice led to a reduction in the milieu of pro-inflammatory cytokines and an induction of anti-inflammatory cytokine like IL-10.

We next studied the skeletal effects of mIL-18BPd treatment. It was observed that Ovx induced micro architectural deterioration was significantly restored by mIL-18BPd treatment. More importantly, sham group treated with mIL-18BPd presented with a better bone micro architectural parameters compared to sham group which strengthens the osteoprotective potential of IL-18BP. Anabolic agents like PTH lead to increase in periosteal apposition^48^. We observed increased cortical thickness and cortical bone area in Ovx mice treated with mIL-18BPd. In agreement with this data, femur biomechanical parameters showed that mIL-18BPd treatment conferred a greater energy required for breaking force compared to Ovx untreated mice. Effect of IL-18BP was also studied on various osteoclasts and osteoblasts functional parameters. Treatment of Ovx mice with mIL-18BPd led to significant reduction in osteoclasts number and surface supporting the anti-osteoclastogenic role of IL-18BP. Moreover, an increase in the number of bone lining cells and serum P1NP, an established bone anabolic marker was observed with mIL-18BPd treatment which might be the reason which led to the increased cortical bone deposition in treated animals compared to untreated ones. IL-18BP also binds to IL-37 cytokine apart from IL-18^36^. Despite the failure to identify an IL-37 isoform in mouse till date^36^, it was important to ascertain that the skeletal and immune effects observed with IL-18BP were due to its inhibition of IL-18. Reduced percentage of IL-17A+ve cells and increased percentage of FoxP3+ve cells in Ovx animals treated with neutralizing antibody against IL-18 supported our observations that IL-18BP is exerting its immunoprotective and osteoprotective effects by binding to IL-18 and preventing its interaction with IL-18 receptor. This data was corroborated with increased mRNA transcripts of osteogenic markers like Runx-2 and BMP-2 in bone tissue and co-culture model of Ovx animals treated with neutralizing IL-18 antibody.

We also evaluated serum IL-18BP and IL-18 levels in post menopausal women. To the best of our knowledge, IL-18BP levels have not been correlated with BMD in postmenopausal women. There are indirect studies where it is reported that Cushing Syndrome patients, which have a high risk of osteoporotic fractures have high IL-18 serum levels^24^. Our results show a significant increase in serum IL-18 levels in osteoporotic post menopausal women while serum hIL-18BPa levels were decreased. Importantly, this decrease in serum hIL-18BPd levels correlated with BMD at lumbar spine (L1–L4) where patients with low BMD also exhibited decreased hIL-18BPd serum levels. Thus, low IL-18BPd levels may be indicative of increased risk of osteoporosis.

**Figure 9:**
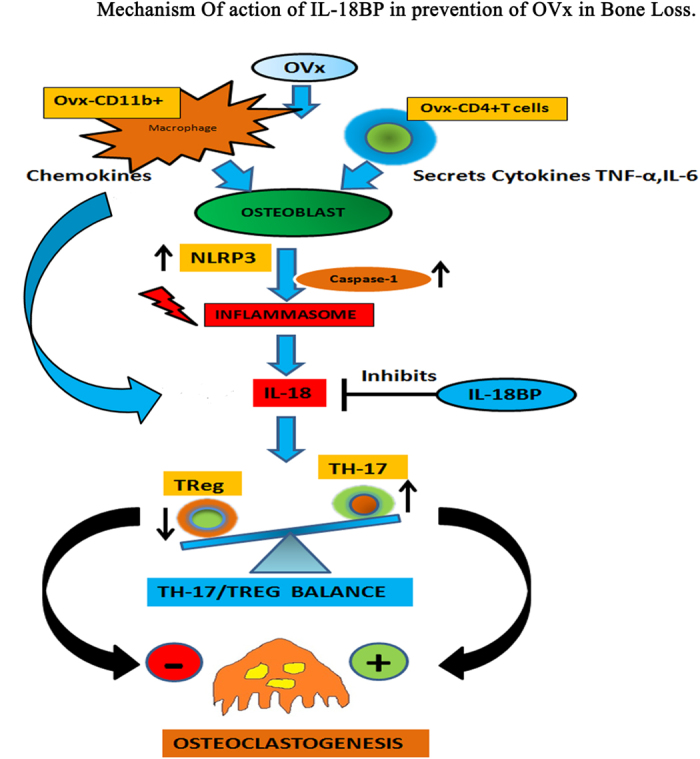
A proposed model depicting role of mIL-18BP in suppressing Ovx induced immunological alterations and bone loss. As a result of Ovx there is increased production of pro-inflammatory cytokine IL-18 which promotes TH17 responses leading to an increase in IL-17 production. IL-17 would then induce osteoclastogenesis and bone loss. This effect is inhibited by IL-18BP which antagonizes IL-18 and reduces TH17 cells thereby promoting Treg cells development which are known to suppress osteoclastogenesis. Simultaneously, mIL-18BP also induces osteoblasts to inhibit NLRP3 inflammasome and Caspase-1 activation which are involved in processing of IL-18 into its active form. The net result is decreased Th17 production, less of IL-17 secretion, inhibition of osteoclastogenesis and thus reduced bone loss.

In conclusion, IL-18BP inhibits inflammasome mediated IL-18 processing and activation which leads to less IL-18 secretion and decreased TH17 differentiation (Fig. 9). This ultimately leads to an increase in Treg/Th17 ratio and an inhibition of osteoclastogenesis and reduced bone loss. This study forms a strong basis for using humanized IL-18BP towards the treatment of postmenopausal osteoporosis.

## Materials and Method

### Reagents and chemicals

Details are given in Supplementary information for material and methods.

### PBMC Isolation

PBMCs were isolated as per the standard protocol. Details are given in Supplementary information for material and methods.

### Flow Cytometry

PBMC cells were labelled with anti CD3, CD4, CD28 and B220 antibodies (APC conjugated anti-mouse CD3, PE-conjugated anti-mouse CD4, FITC conjugated anti-mouse CD28 and APC conjugated anti B220 antibodies) to assess the percentage of CD4+, CD4+CD28+ in CD3+ cells and B220+ cells as per previously published protocol. Details are given in Supplementary information for material and methods.

### Intracellular staining

Intracellular staining was performed as per manufacturer’s instructions for IL-17A cytokine and Treg transcription factor Foxp3. Details are given in Supplementary information for material and methods.

### Cytometric Bead Array flex

Cytometric Bead Array (CBA) (BD biosciences, San Diego, CA, USA) was used for the measurement of various cytokines in different *in vivo* groups. Details are given in Supplementary information for material and methods.

### Total RNA isolation and Quantitative Real time PCR

Total RNA was isolated from PBMCs, Osteoblasts, CD4+, CD11b+ and B220+ cells in all *in vitro* and *in vivo* groups using Trizol (Invitrogen Carlsband CA,USA). Details are given in Supplementary information for material and methods. Primer sequences are given in Supplementary Table 2.

### *In vivo* studies

For animal study 8–9 weeks balb/c mice were used for *in vivo* experiments. The study was conducted in accordance with current legislation on animal experiments and was approved by institutional ethics committee, Central Drug Research Institute (CPSCEA registration no. 34/1999, dated November 3, 1999, extended to 2015, approval reference no. IAEC/2013/93/renew01, dated December 03, 2014). All methods involving humans were performed in accordance with the relevant guidelines and regulations and was approved by Institutional Ethics Committee, CSIR-CDRI (approval no. CDRI/IEC/2015/A9) and Sanjay Gandhi Post Graduate Institute of Medical Sciences, Lucknow (IEC Code 2014-21-EMP-74 Dated 16th July 2015). All the subjects were informed of details of the experiment prior to obtaining a sample of peripheral venous blood. A written informed consent document was obtained from each participant. Details are given in Supplementary information for material and methods.

### Co-Culture of osteoblast with T cells and Macrophages

Adult mice 8–10 weeks were ovariectomized and left for one month. After one month time period CD4+ T cells and CD11b+ macrophages were isolated by using micro beads and columns with MACS cells isolator from Miltenyi-biotech. On previous day mouse calvarial osteoblasts were cultured.

Mouse calvarial osteoblasts were seeded for 12 hours in T-25. After attachment of osteoblasts, cells were co cultured with Ovx T cells or CD11b+ macrophages in the ratio of 1:1. One set of co-culture was treated with IL-18BPd (100 ng/ml) for 48 hours. After 48 hours media was discarded and flask were washed with PBS. After this 1 ml of Trizol was added to the flasks and mouse osteoblast cells were harvested for RNA collection. RNA was isolated and cDNA was synthesized for checking the expression of various genes by RT-PCR.

### μCT Scanning, bone strength testing and Histomorphometric analysis

Microcomputed tomographic (μCT) determination of excised bones was carried out using previously published protocol^10^,^27^,^29^,^49^. Bone mechanical strength was examined by 3 point bending strength of femur mid-diaphysis as reported earlier^49^,^50^. Histomorphometric analyses were conducted as reported earlier^49^. Details are given in Supplementary information for material and methods.

### BMD analysis by DXA

BMD of post menopausal women (age 50 years and above) was measured by Dual Energy X-Ray Absorptiometry (DXA, Hologic QDR Discovery, Bedford, USA). Subjects with intake of glucocorticoids, strontium renelate, bisphosphonates, teriparatide, pharamacologic dose of vitamin D, vitamin D analogs, prolonged immobility, malignancy, chronic renal or hepatic faliure, untreated hypo- or hyperthyroidism, and primary hyperparathyroidism were excluded. Informed consent was obtained from all subjects. Based on WHO criteria of densitometric definition of osteoporosis, postmenopausal women were categorized into three groups; normal, osteopenic and osteoporotic. Osteoporosis was defined as T score ≤−2.5, subjects with T score between −2.5 to −1 were defined as osteopenic and those with T score above −1 were considered as normal group.

### Serum ELISAs

Mice serum levels of Type I collagen N-terminal pro-peptide (P1NP) and IL-18 were determined by ELISA kits purchased from Immunodiagnostic Systems Inc. UK and Ray Biotech (Norcross, GA, USA) respectively by following the manufacturer’s protocols. Levels of IL-18BPa and IL18 in human serum sample were analyzed in normal, osteopenic and osteoporotic subjects by Elisa kits from Ray Biotech (Norcross, GA, USA).

### Determination of Bone lining cells

Details are given in Supplementary information for material and methods.

### Statistical Analysis

Data are expressed as mean ± SE. The data obtained in the experiment were subjected to one way ANOVA followed by Newman–Keuls test of significance using Prism version 3.0 software. Qualitative observations have been represented following assessment made by three individuals blinded to the experimental design. For human subjects Kruskal-Wallis test was used for statistical analysis followed by Dunn’s Multiple comparison test. Data analysis was done using Graph Pad prism version 5.0 software. Student’s t- test was used to compare between two means. Correlation between variables was performed using Pearsons’ correlation test. Values of p < 0.05 were considered significant.

## Additional Information

**How to cite this article**: Mansoori, M. N. *et al*. IL-18BP is decreased in osteoporotic women: Prevents Inflammasome mediated IL-18 Activation and reduces Th17 differentiation. *Sci. Rep.* **6**, 33680; doi: 10.1038/srep33680 (2016).

## Supplementary Material

Supplementary Information

## References

[b1] Clowes J. A., Riggs B. L. & Khosla S. The role of the immune system in the pathophysiology of osteoporosis. Immunol Rev 208, 207–227 (2005). 16313351 10.1111/j.0105-2896.2005.00334.x

[b2] Picherit C. . Soybean isoflavones dose-dependently reduce bone turnover but do not reverse established osteopenia in adult ovariectomized rats. J Nutr 131, 723–728 (2001). 11238750 10.1093/jn/131.3.723

[b3] Hardy R. & Cooper M. S. Bone loss in inflammatory disorders. J Endocrinol 201, 309–320 (2009). 19443863 10.1677/JOE-08-0568

[b4] Kanis J. A. & McCloskey E. V. Bone turnover and biochemical markers in malignancy. Cancer 80, 1538–1545 (1997). 9362420 10.1002/(sici)1097-0142(19971015)80:8+<1538::aid-cncr3>3.3.co;2-v

[b5] Kular J., Tickner J., Chim S. M. & Xu J. An overview of the regulation of bone remodelling at the cellular level. Clin Biochem 45, 863–873 (2012). 22465238 10.1016/j.clinbiochem.2012.03.021

[b6] Paiva K. B. & Granjeiro J. M. Bone tissue remodeling and development: Focus on matrix metalloproteinase functions. Arch Biochem Biophys 561C, 74–87 (2014). 25157440 10.1016/j.abb.2014.07.034

[b7] Mori G., D’Amelio P., Faccio R. & Brunetti G. The Interplay between the bone and the immune system. Clin Dev Immunol 2013, 720504 (2013). 23935650 10.1155/2013/720504 PMC3725924

[b8] Zupan J., Jeras M. & Marc J. Osteoimmunology and the influence of pro-inflammatory cytokines on osteoclasts. Biochem Med (Zagreb) 23, 43–63 (2013). 23457765 10.11613/BM.2013.007 PMC3900089

[b9] Kitazawa R., Kimble R. B., Vannice J. L., Kung V. T. & Pacifici R. Interleukin-1 receptor antagonist and tumor necrosis factor binding protein decrease osteoclast formation and bone resorption in ovariectomized mice. J Clin Invest 94, 2397–2406 (1994). 7989596 10.1172/JCI117606 PMC330070

[b10] Tyagi A. M. . Estrogen deficiency induces the differentiation of IL-17 secreting Th17 cells: a new candidate in the pathogenesis of osteoporosis. Plos One 7, e44552 (2012). 22970248 10.1371/journal.pone.0044552 PMC3438183

[b11] Kimble R. B., Bain S. & Pacifici R. The functional block of TNF but not of IL-6 prevents bone loss in ovariectomized mice. J Bone Miner Res 12, 935–941 (1997). 9169353 10.1359/jbmr.1997.12.6.935

[b12] Conforti-Andreoni C. . Uric acid-driven Th17 differentiation requires inflammasome-derived IL-1 and IL-18. J Immunol 187, 5842–5850 (2011). 22058415 10.4049/jimmunol.1101408

[b13] Mills K. H., Dungan L. S., Jones S. A. & Harris J. The role of inflammasome-derived IL-1 in driving IL-17 responses. J Leukoc Biol 93, 489–497 (2013). 23271701 10.1189/jlb.1012543

[b14] Nakanishi K., Yoshimoto T., Tsutsui H. & Okamura H. Interleukin-18 regulates both Th1 and Th2 responses. Annu Rev Immunol 19, 423–474 (2001). 11244043 10.1146/annurev.immunol.19.1.423

[b15] Banda N. K. . Mechanisms of inhibition of collagen-induced arthritis by murine IL-18 binding protein. J Immunol 170, 2100–2105 (2003). 12574381 10.4049/jimmunol.170.4.2100

[b16] Dinarello C. A., Novick D., Kim S. & Kaplanski G. Interleukin-18 and IL-18 binding protein. Front Immunol 4, 289 (2013). 24115947 10.3389/fimmu.2013.00289 PMC3792554

[b17] Zhang W. . IL-18 upregulates the production of key regulators of osteoclastogenesis from fibroblast-like synoviocytes in rheumatoid arthritis. Inflammation 36, 103–109 (2013). 22945280 10.1007/s10753-012-9524-8

[b18] Matsui K., Tsutsui H. & Nakanishi K. Pathophysiological roles for IL-18 in inflammatory arthritis. Expert Opin Ther Targets 7, 701–724 (2003). 14640907 10.1517/14728222.7.6.701

[b19] Gracie J. A. . A proinflammatory role for IL-18 in rheumatoid arthritis. J Clin Invest 104, 1393–1401 (1999). 10562301 10.1172/JCI7317 PMC409841

[b20] Carbotti G. . The IL-18 antagonist IL-18-binding protein is produced in the human ovarian cancer microenvironment. Clinical cancer research: an official journal of the American Association for Cancer Research 19, 4611–4620, 10.1158/1078-0432.CCR-13-0568 (2013). 23873689

[b21] Lee S. . Development of isoform-specific monoclonal antibodies against human IL-18 binding protein. Hybridoma (Larchmt) 29, 517–524 (2010). 21087098 10.1089/hyb.2010.0058

[b22] McInnes I. B. & Liew F. Y. Cytokine networks–towards new therapies for rheumatoid arthritis. Nat Clin Pract Rheumatol 1, 31–39 (2005). 16932625 10.1038/ncprheum0020

[b23] Tak P. P., Bacchi M. & Bertolino M. Pharmacokinetics of IL-18 binding protein in healthy volunteers and subjects with rheumatoid arthritis or plaque psoriasis. Eur J Drug Metab Pharmacokinet 31, 109–116 (2006). 16898079 10.1007/BF03191127

[b24] Kristo C. . Raised serum levels of interleukin-8 and interleukin-18 in relation to bone metabolism in endogenous Cushing’s syndrome. Eur J Endocrinol 146, 389–395 (2002). 11888846 10.1530/eje.0.1460389

[b25] Zhu L. . Activation of NALP1 inflammasomes in rats with adjuvant arthritis; a novel therapeutic target of carboxyamidotriazole in a model of rheumatoid arthritis. British journal of pharmacology 172, 3446–3459, 10.1111/bph.13138 (2015). 25799914 PMC4500378

[b26] Youm Y. H. . The Nlrp3 inflammasome promotes age-related thymic demise and immunosenescence. Cell reports 1, 56–68, 10.1016/j.celrep.2011.11.005 (2012). 22832107 PMC3883512

[b27] Tyagi A. M. . Premature T cell senescence in Ovx mice is inhibited by repletion of estrogen and medicarpin: a possible mechanism for alleviating bone loss. Osteoporos Int 23, 1151–1161 (2012). 21562872 10.1007/s00198-011-1650-x

[b28] Kumar H. . Involvement of the NLRP3 inflammasome in innate and humoral adaptive immune responses to fungal beta-glucan. Journal of immunology 183, 8061–8067, 10.4049/jimmunol.0902477 (2009). 20007575

[b29] Tyagi A. M. . Daidzein prevents the increase in CD4+ CD28null T cells and B lymphopoesis in ovariectomized mice: a key mechanism for anti-osteoclastogenic effect. Plos One 6, e21216 (2011). 21731677 10.1371/journal.pone.0021216 PMC3120851

[b30] Yang X. O. . Molecular antagonism and plasticity of regulatory and inflammatory T cell programs. Immunity 29, 44–56 (2008). 18585065 10.1016/j.immuni.2008.05.007 PMC2630532

[b31] Dong C. TH17 cells in development: an updated view of their molecular identity and genetic programming. Nat Rev Immunol 8, 337–348 (2008). 18408735 10.1038/nri2295

[b32] Yoo J. K. . IL-18 induces monocyte chemotactic protein-1 production in macrophages through the phosphatidylinositol 3-kinase/Akt and MEK/ERK1/2 pathways. J Immunol 175, 8280–8286 (2005). 16339568 10.4049/jimmunol.175.12.8280

[b33] Ginaldi L., Di Benedetto M. C. & De Martinis M. Osteoporosis, inflammation and ageing. Immun Ageing 2, 14 (2005). 16271143 10.1186/1742-4933-2-14 PMC1308846

[b34] Dobnig H. & Turner R. T. Evidence that intermittent treatment with parathyroid hormone increases bone formation in adult rats by activation of bone lining cells. Endocrinology 136, 3632–3638 (1995). 7628403 10.1210/endo.136.8.7628403

[b35] Hale L. V. . PINP: a serum biomarker of bone formation in the rat. Bone 40, 1103–1109 (2007). 17258520 10.1016/j.bone.2006.11.027

[b36] Ji Q. . Elevated plasma IL-37, IL-18, and IL-18BP concentrations in patients with acute coronary syndrome. Mediators of inflammation 2014, 165742, 10.1155/2014/165742 (2014). 24733959 PMC3964842

[b37] Sivakumar P. V. . Interleukin 18 is a primary mediator of the inflammation associated with dextran sulphate sodium induced colitis: blocking interleukin 18 attenuates intestinal damage. Gut 50, 812–820 (2002). 12010883 10.1136/gut.50.6.812 PMC1773244

[b38] Dinarello C. A. Interleukin 1 and interleukin 18 as mediators of inflammation and the aging process. Am J Clin Nutr 83, 447S–455S (2006). 16470011 10.1093/ajcn/83.2.447S

[b39] Makiishi-Shimobayashi C. . Interleukin-18 up-regulates osteoprotegerin expression in stromal/osteoblastic cells. Biochem Biophys Res Commun 281, 361–366 (2001). 11181055 10.1006/bbrc.2001.4380

[b40] Zhong X., Wang H. & Huang S. Endothelin-1 induces interleukin-18 expression in human osteoblasts. Arch Oral Biol 59, 289–296 (2014). 24581851 10.1016/j.archoralbio.2013.11.006

[b41] Udagawa N. . Interleukin-18 (interferon-gamma-inducing factor) is produced by osteoblasts and acts via granulocyte/macrophage colony-stimulating factor and not via interferon-gamma to inhibit osteoclast formation. J Exp Med 185, 1005–1012 (1997). 9091574 10.1084/jem.185.6.1005 PMC2196233

[b42] Dai S. M., Nishioka K. & Yudoh K. Interleukin (IL) 18 stimulates osteoclast formation through synovial T cells in rheumatoid arthritis: comparison with IL1 beta and tumour necrosis factor alpha. Ann Rheum Dis 63, 1379–1386 (2004). 15479886 10.1136/ard.2003.018481 PMC1754791

[b43] Bonar S. L. . Constitutively activated NLRP3 inflammasome causes inflammation and abnormal skeletal development in mice. PloS one 7, e35979, 10.1371/journal.pone.0035979 (2012). 22558291 PMC3338787

[b44] Murakami Y., Otsuki M., Kusumoto K., Takeuchi S. & Takahashi S. Estrogen inhibits interleukin-18 mRNA expression in the mouse uterus. The Journal of reproduction and development 51, 639–647 (2005). 16034191 10.1262/jrd.17029

[b45] Xu Y. . NLRP3 inflammasome activation mediates estrogen deficiency-induced depression- and anxiety-like behavior and hippocampal inflammation in mice. Brain, behavior, and immunity, 10.1016/j.bbi.2016.02.022 (2016). 26928197

[b46] Netea M. G., Kullberg B. J. & Verschueren I. & Van Der Meer, J. W. Interleukin-18 induces production of proinflammatory cytokines in mice: no intermediate role for the cytokines of the tumor necrosis factor family and interleukin-1beta. European journal of immunology 30, 3057–3060, 10.1002/1521-4141(200010)30:10<3057::AID-IMMU3057>3.0.CO;2-P (2000). 11069090

[b47] van de Veerdonk F. L. . The inflammasome drives protective Th1 and Th17 cellular responses in disseminated candidiasis. European journal of immunology 41, 2260–2268, 10.1002/eji.201041226 (2011). 21681738 PMC3939807

[b48] Silva B. C. & Bilezikian J. P. Parathyroid hormone: anabolic and catabolic actions on the skeleton. Current opinion in pharmacology 22, 41–50, 10.1016/j.coph.2015.03.005 (2015). 25854704 PMC5407089

[b49] Tyagi A. M. . Enhanced immunoprotective effects by anti-IL-17 antibody translates to improved skeletal parameters under estrogen deficiency compared with anti-RANKL and anti-TNF-alpha antibodies. J Bone Miner Res 29 (2014). 10.1002/jbmr.2228 24677326

[b50] Mansoori Mohd N. . “Methoxyisoflavones formononetin and isoformononetin inhibit the differentiation of Th17 cells and B-cell lymphopoesis to promote osteogenesis in estrogen-deficient bone loss conditions”. Menopause 23.5, 565–576 (2016). 27070807 10.1097/GME.0000000000000646

