# Supplementary material for: IL-18BP is decreased in osteoporotic women: Prevents Inflammasome mediated IL-18 activation and reduces Th17 differentiation
Source: Sci Rep. 2016 Sep 21;6:33680. doi: 10.1038/srep33680 (PMC5030484; doi:10.1038/srep33680)
Supplement: Supplementary Information [file srep33680-s1.pdf]

IL-18BP is decreased in osteoporotic women: Prevents Inflammasome mediated IL-18 activation and reduces Th17 differentiation

Mohd Nizam Mansoori[1], Priyanka Shukla[1], Manisha Kakaji[2] , Abdul M Tyagi[1], Kamini Srivastava , Manoj Shukla[2], Manisha Dixit[1], Jyoti Kureel[1], Sushil Gupta[2] and Divya Singh[1*]

1.Division of Endocrinology and Centre for Research in Anabolic Skeletal Targets in Health and Illness (ASTHI), CSIR-Central Drug Research Institute, B.S. 10/1, Sector-10, Jankipuram Extension, , Lucknow, India.

2. Department of Endocrinology, Sanjay Gandhi Postgraduate Institute of Medical Sciences, Lucknow,India.

[*]**Corresponding author:** Divya Singh

Tel: +91-522 2772550 Ext: 4395; Fax: +91-522 2771940

E-mail address: divya_singh@cdri.res.in

Reprints to be addressed to: Divya Singh

Running title: IL-18BP is decreased in osteoporotic patients

Supporting grants: Centre for Research in Anabolic Skeletal Targets in Health and Illness (ASTHI), Council of Scientific and Industrial Research (CSIR), and Department of Biotechnology, Government of India.

Supplementary material and methods:

Reagents and chemicals:

All the cell culture chemicals and primers were purchased from sigma-Aldrich (St Louis, MO). IL-18 cytokine was purchased from Prospec, Ness-Ziona, Israel. mIL-18BPd was purchased from leinco technologies, Inc. CBA flex kit was purchased from BD Biosciences

(Mississauga,ON,CA) anti-IL-18 antibody was purchased from abcam (Cambridge ,UK) PINP Elisa kit was purchased from Immunodiagnostics system (Ltd UK) and IL-18 Elisa kit was purchased from Ray biotech. FACS antibodies like CD4-PE, CD28-FITC, IL-17A-PE, IgM-PE and B220+FITC were purchased from Biolegend Nu life. cDNA synthesis kit was purchased from Invitrogen (Carlsbad, CA, USA).

PBMC Isolation:

After autopsy long bones and spleen were flushed out with the help of PBS. Cells were washed and mixed with PBS. After this cell suspension was loaded on hisep media and centrifuged at 400g for 30 minutes at 25ºC. PBMC ring was isolated carefully and cells were washed twice with PBS. Half of the PBMCs were used for FACS staining and rest of the PBMCs for Isolation of CD4+ T cells, B220+ cells, CD11b+ macrophage cells.

Flow Cytometry

PBMC cells were labelled with anti-CD4, CD28 and B220 antibodies (PE-conjugated anti-mouse CD4, FITC conjugated anti-mouse CD28, PE conjugated IgM and APC conjugated anti B220 antibodies) to assess the percentage of CD4 +, CD4+CD28+ cells and B220+ cells as per previously published protocol. Specificity of immunostaining was ascertained by the background fluorescence of cells incubated with Ig isotype controls. Fluorescence data from at least 10,000 cells were collected from each sample. Immunostaining was done as per manufacturer's instructions. In brief, single cell suspension of the BM was prepared in PBS. Cells were counted using haemocytometer and were re-suspended in FACS buffer as 10^6 cells/500 µl PBS and antibody was added as 10 µl/10^6 cells and further incubated for 45 minutes at room temperature. After incubation cells were washed twice with PBS and transferred to FACS tubes for analysis. FACS Caliber and FACS Aria (BD Biosciences

Mississauga, ON, CA) were used to quantify the percentage of CD4+, CD4+CD28+ T cells i and B220+ B cells in all the groups.

Intracellular staining:

Intracellular staining was performed as per manufacturer's instructions for IL-17A cytokine and Treg transcription factor Foxp3. Treg staining kit was purchased from Biolegend San Diego CA, USA. For Staining IL-17A, PBMCs were isolated from spleenocytes and these cells were stimulated for 6h with 10ng/ml PMA , 250 ng/ml ionomycin and 10μg/ml bredfeldin A. After this cells were harvested, fixed and permeabilized by using Leucoperm Reagent A (Fixation Reagent) and Leucoperm Reagent B (permeabilizing reagent). Permeabilization was followed by intracellular staining of IL-17A cytokine with PE labelled IL-17 antibody. Specificity of immunostaining was ascertained by back ground flourecence of the cells incubated by Isotype control of IgG. After intracellular staining, cells were washed twice with PBS and transferred in FACS tube for analysis. FACS calibur and FACS Aria (BD Biosciences Mississunga, ON, CA) were used to quantify % of IL-17A positive cells. Also, Percentage of CD4+CD25+ cells and CD25+FOXP3+ cells was measured by using FACS Aria (BD Biosciences San Diego,USA).

Cytometric Bead Array:

Cytometric Bead Array (CBA) flex from BD biosciences was used for the measurement of various levels of cytokines in different *in vivo* groups. Levels of IL-6, TNF-α, IL-2, IFN-γ, IL-17A and IL-10 were measured in serum samples using Fluorescent bead based technology according to Manufacturer's instruction (BD biosciences, San Diego, USA).Fluorescent signals were read and analyzed on FACS Aria Flow cytometer (BD biosciences, San Diego, USA) with help of BD FCAP Array v.1.0.1 software BD Biosciences.

Total RNA isolation and Quantitative Real time PCR:

Total RNA was isolated from PBMCs, osteoblast, CD4+ and CD11b+ cells in all *in vitro* and *in vivo* groups using Trizol (Invitrogen Carlsbad, CA, USA). cDNA was synthesized from 1μg of total RNA with Revert AidTM H Minus first strand cDNA synthesis kit (Fermentas, Mumbai, India). SYBER green chemistry was used for quantitative determination of mRNAs of IL-18BPd, IL-18, BMP-2, TNF-α, IL-6, Foxp3, ROR-α ,ROR-γt,Runx-2,NLRP3,Caspase-1,TNF-α ,Wnt-10B,IL-18BPa(human) and housekeeping gene GAPDH.

Primers for different genes were designed uising universal probe library. Sequences of primers are provided in supplementary table 1. For real time cDNA was amplified with lightcycler 480 (Roche Diagnoastics Pvt. Ltd., Basel, Switzerland) to allow for quantitative detection of product in a 20 μl reaction volume. The temperature of reaction was 95ºC for 5min,40 cycles of denaturation at 94ºC for 2 minutes annealing and extension at 62 ºC for 30 sec, extension at 72 ºC for 30 sec. GAPDH was used to normalize differences in RNA isolation, RNA degradation and efficiencies of Reverse transcription.

***In Vivo* studies:**

For animal Study 8-9 weeks balb/c mice were used for in vivo experiments. The study was conducted in accordance with current legislation on animal experiments and was approved by institutional ethics committee, Central Drug Research Institute (CPSCEA registration no.34/1999, dated November 3, 1999, extended to 2015, approval reference no. IAEC/2013/93/renew01, dated December 03, 2014). All the animals were housed at 25ºC, 12 hour light and 12 hour dark cycles. Normal chow diet and water were provided ad libtimum. Mice were divided into four groups with each group containing 10 mice. The groups in experiment were sham operated (ovary intact) mice, sham + IL-18BPd (0.5 mg/kg ovariectomized (Ovx) mice and Ovx mice + IL-18BP (0.5mg/kg body weight) Treatment was

continued for a period of 4 week with s.c. injection twice weekly. After one month, animals were sacrificed; long bones and serum samples were collected from different groups. Bones were cleaned and bone marrow was flushed out from bone marrow. PBMC was isolated by using hisep LSM (1084) himedia by means of density (1.084±0010) from bone marrow. These cells were used for FACS Staining and for isolation of CD4+T cells, CD8+T cells, B220+ B cells and CD11b+ macrophage cells by microbeads isolation from MACS separator (Milteny Biotech,Singapore). All methods involving humans were performed in accordance with the relevant guidelines and regulations and were approved by Institutional Ethics Committee, CSIR-CDRI (approval no.CDRI/IEC/2015/A9) and Sanjay Gandhi Post Graduate Institute of Medical Sciences, Lucknow (IEC Code 2014-21-EMP-74 Dated 16th July 2015). All the subjects were informed of details of the experiment prior to obtaining a sample of peripheral venous blood. A written informed consent document was obtained from each participant.

µCT Scanning, bone strength testing and Histomorphometric analysis

Microcomputed tomographic (µCT) determination of excised bones was carried out using Sky Scan 1076 CT Scanner (Aartselaar, Belgium) using previously published protocol(2-5). Three samples were scanned per batch at nominal resolution pixcel 18 µm .Reconstruction was carried out using a modified Feldkamp algorthim using the Sky Scan Nerocon Software, which facilitates network distributed reconstruction carried on personal computer running simultaneously. The x ray source was set at 70kV and 100mA, with a pixel size of 18 µm. To analyze trabecular region, region of interest (ROI) was drawn at a total of 100 slices in the region of secondary spongiosa situated at 1.5mm from distal border of growth plate, excluding all primary spongiosa and cortical bone. For cortical bone analysis, 350 serial image slides were discarded from growth plate to exclude any trabecular region, and 100 consecutive image slides were selected and quantification was done using CTAN software.

Bone mechanical strength was examined by 3 point bending strength of femur mid-diaphysis using Bone strength tester model TK252C as reported earlier (2). Graphs were plotted against stiffness (N/m), Power (N) and Energy (mJ) for different groups. Histomorphometric analyses were conducted using Bioquant Image Analysis software (Bioquant) as reported earlier (2). TRAP (tartrate-resistant acid phosphatase) staining of osteoclasts was performed using a leukocyte acid phosphatase staining kit (Sigma, St.Louis MO,USA).

Determination of Bone lining cells

Deparaffinized and hydrated femoral epiphysis sections of different groups were immersed in Mayer's hematoxylin for 1 minute, washed with running tap water for 15 minutes and incubated in 1% eosin aqueous solution for 1-2 minutes at room temperature. Following this, the sections were dehydrated with ascending concentrations of ethanol solutions, cleared with xylene and mounted with a cover slip using DPX (Sigma St. Louis MO, USA) mounting medium. Lining cells on the periosteal bone surface were calculated with Image-Pro Plus 6.1 software.

Statistical Analysis:

Data are expressed as mean ± SE. The data obtained in the experiment were subjected to one way ANOVA followed by Newman–Keuls test of significance using Prism version 3.0 software. Qualitative observations have been represented following assessment made by three individuals blinded to the experimental design. For human subjects Kruskal-Wallis test was used for statistical analysis followed by Dunns Multiple comparison test. Data analysis was done using Graph Pad prism version 5.0 software. Student's t- test was used to compare

between two means .Correlation between variables was performed using Pearsons' correlation test values of p<0.05 were considered significant.

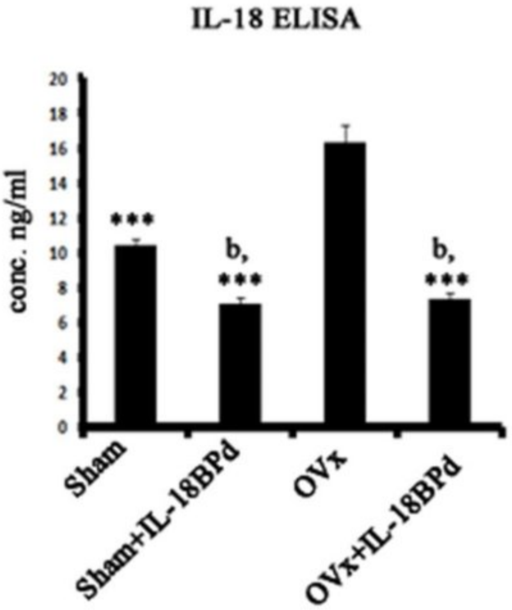

Supplementary Fig S1: Effect of mIL-18BPd on serum level of IL-18. Serum level of IL-18 in Ovx group was increased in comparison with sham group.IL-18BP treatment reversed these effects. Serum level of IL-18 in sham +mIL-18BPd was also decreased when compared with sham group. n=10 mice/group; data are presented as mean ± SE; ***$p<0.001$ compared with Ovx group; **$p<0.01$ compared with Ovx group; *$p<0.05$ compared with Ovx; $^{a}p<0.05$ compared with sham group; $^{b}p<0.01$ compared with sham group.

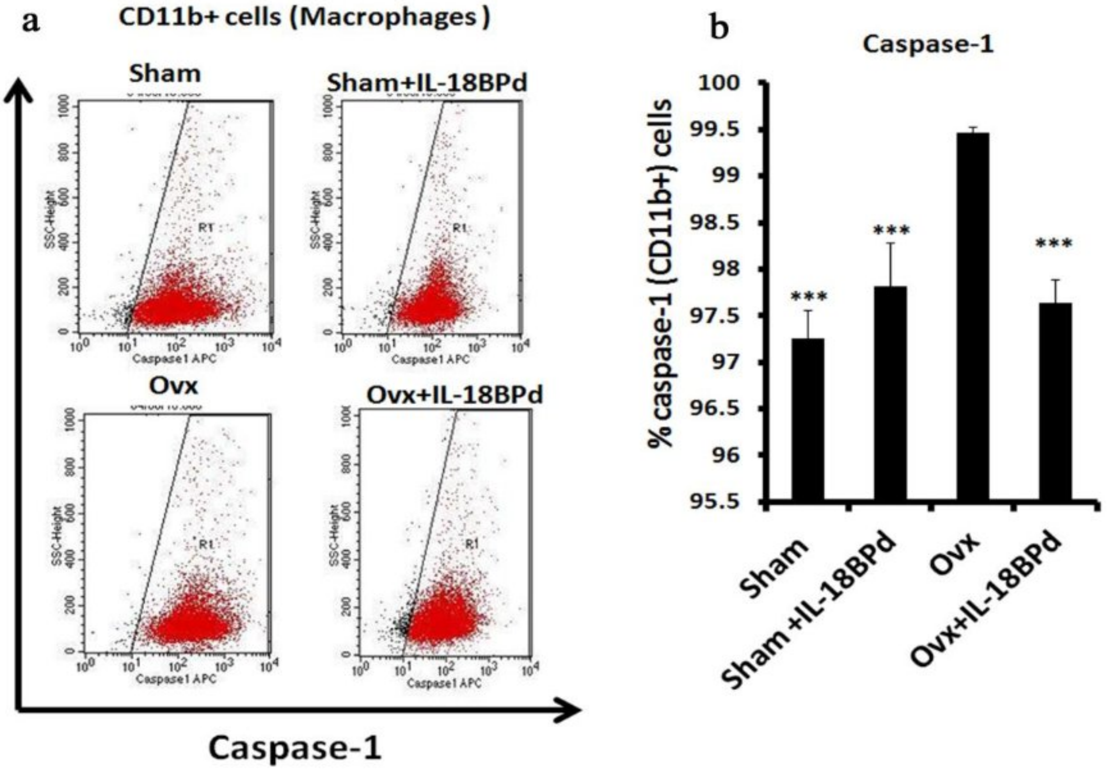

Supplementary Fig S2: Effect of mIL-18BPd caspase-1 expression in CD11b+(Macrophages cells) by FACS. Caspase-1 expression was increased in macrophages isolated from Ovx mice. Treatment with IL-18BPd in Ovx macrophages has significantly decreased the expression of caspase-1.while caspase-1 expression has no significant difference between treated and untreated macrophages from sham group. n=8 mice/group; data are presented as mean ± SE; ***p<0.001 compared with Ovx group.

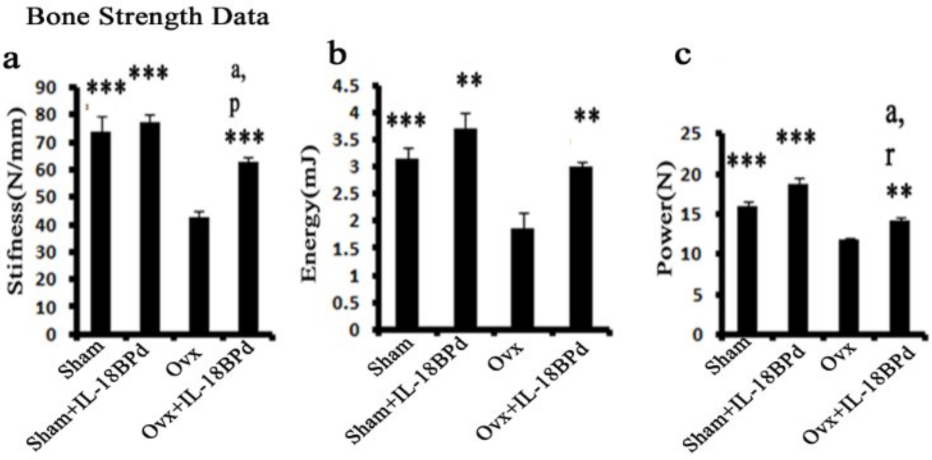

Supplementary Fig S3: Effect of mIL-18BPd on bone biomechanical properties.Bone strength parameters like (a) Stiffness (b) Energy and (c) Power were reduced in the Ovx, and mIL-18BP treatment reversed these effects. n=10 mice/group; data are presented as mean ± SE; ***$p<0.001$ compared with Ovx group; **$p<0.01$ compared with Ovx group; *$p<0.05$ compared with Ovx; [a]$p<0.05$ compared with sham group; [b]$p<0.01$ compared with sham group;[q]$p<0.01$ compared with sham+mIL-18BPd.

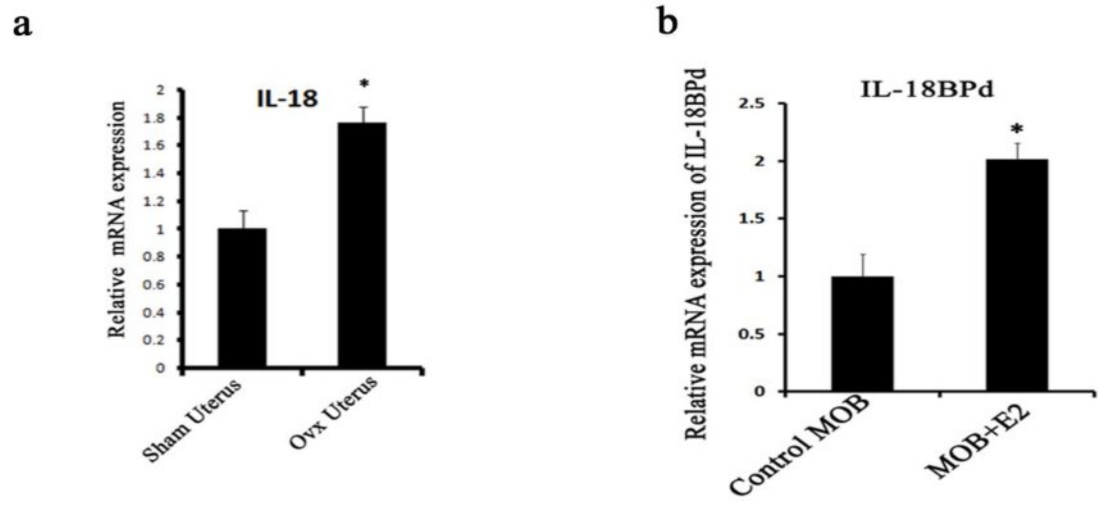

Supplementary Fig S4: (a) mRNA expression of IL-18 in Sham and Ovx uterus.IL-18 expression is increased in Ovx uterus.n=10 mice/group; data are presented as mean ± SE; *p<0.05 compared with sham group.(b)Effect of E2 on mice osteoblasts. Treatment with E2 has increased the expression of IL-18BPd in mice osteoblasts Data represent three independent experiments and expressed as mean ± SE with 95% confidence interval. Student's t- test was used to compare between two means using Prism version 3.0 software. *P<0.05 compared with control group.

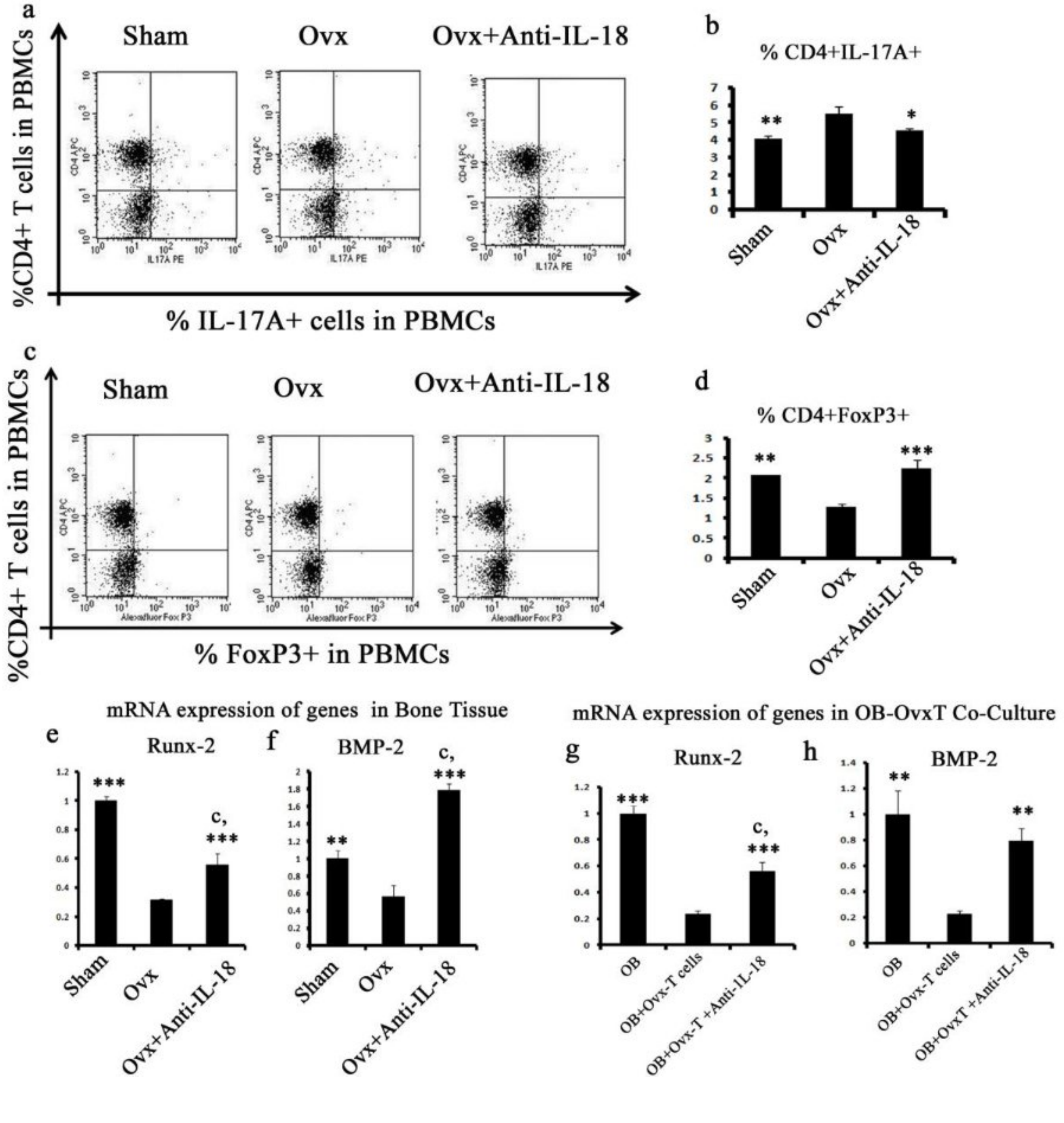

Supplementary Fig S5: Effect of anti-IL-18 on Th17/Treg balance studied.(a) Ovx induces the proliferation of Th17 cells while administration of anti-IL-18 reduces the number of Th-17 cells. (b) Quantification of the FACS data. Percentage of Th17 cells was higher in Ovx animals and treatment with anti-IL-18 inhibited this increase. (c and d) Percentage of CD4+FoxP3+ cells was significantly lower in Ovx group animals. Treatment of Ovx mice with anti-IL-18 led to significant increase in CD4+FoxP3+ Treg cells.(e and f) mRNA expression of Runx-2 and BMP-2 was decreased in Ovx bone while treatment with anti-IL-18 prevented this decrease.(g and h) Co-culture of Ovx-T cells with osteoblast decreases the expression of Runx-2 and BMP-2.Treatment with anti-IL-18 inhibited this effect. n=10 mice/group: data are presented as mean ± SE; ***p<0.001 compared with Ovx group; **p<0.01 compared with Ovx group; *p<0.05 compared with Ovx;

Tibia MicroCT Data

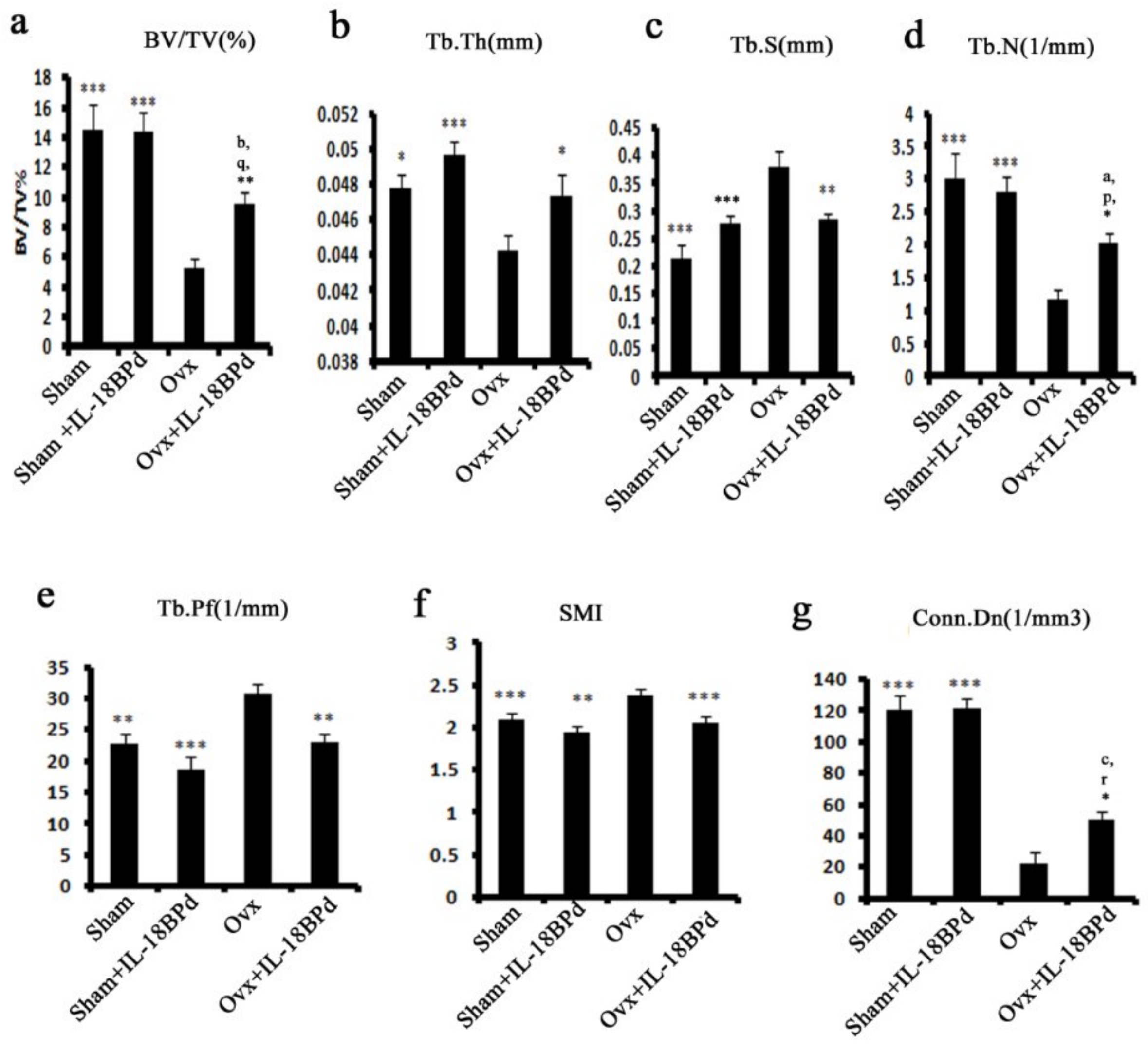

Supplementary Fig S6: Effect of mIL-18BP on Ovx induced bone loss and deterioration of trabecular microarchitecture in Tibia bone. (a) BV/TV, (b) Tb.Th., (c) Tb.S, (d) Tb.N, (e) Tb.Pf, (f) SMI and (g) Conn. D. Administration of mIL-18BP restores Ovx induced alterations in trabecular region of Tibia. Statistical analysis was performed by ANOVA method followed by the Newman–Keuls test of significance using Prism version 3.0 software. n=10 mice/group; data are presented as mean ± SE; ***$p<0.001$ compared with Ovx group; **$p<0.01$ compared with Ovx group; *$p<0.05$ compared with Ovx group; $^{c}p<0.001$ compared with sham+mIL-18BPd group; $^{b}p<0.01$ compared with sham+mIL-18BPd; $^{a}p<0.05$ compared with sham+mIL-18BPd group ; $^{b}p<0.01$ compared with sham+mIL-18BPd group; $^{p}P<0.05$ compared with sham ; $^{q}P<0.01$ compared with sham and $^{r}P<0.001$ compared with sham

Supplementary Table 1: Effect of IL-18BPd on expression of various cytokines.

CYTOKINES	Sham	Sham+IL18BPd	OVx	OVx+IL-18BPd
**IL-6**	7.895±0.68*	5.716±0.49***	10.35±0.55	6.045±0.80***
**TNF-α**	25.11±0.39**	13.84±0.90***,z	29.35±0.22	17.64±1.36***,c,q
**IL-2**	6.432±0.77*	5.95±0.84*,	8.8±0.28	6.58±0.40*,
**IFN-γ**	25.29±1.60**	8.44±0.69***,z	30.37±0.89	8.605±1.02***,c
**IL-17A**	5.56±0.69**	4.5±0.357***	8.62±0.42	4.83±0.91**
**IL-10**	23.84±2.16*	26.07±3.74*	15.97±2.50	27.85±1.35**

Data are mean±SE;

***p<0.001
**p<0.01,
*p<0.05 compared with Ovx group;

cp<0.001 compared with Sham group;

qp<0.01 compared with Sham+IL-18BPd group and

^{z}p<0.001 compared between Sham and Sham+IL-18BPd group

Supplementary Table 2 Sequences of real time PCR primers.

Gene Name	**Primer Sequence**	**Accession Number**
GAPDH	P1-5’-AGCTTGTCATCAACGGGAAG-3’	NM_008084.2
	P2-5’TTTGATGTTAGTGGGGTCTCG-3’	
CASPASE-1	P1-5’CGTGAAAGTGAAAGAAAATCTCAC-3’	NM_009807.2
	P2-GTACTGTCAGAAGTCTTGTGCTCTG-3’	
NLRP3	P1-5’GAATTCCGGCCTTACTTCAA-3’	NM_145827.3
	P2-5‘GGTGTGTGAAGTTCTGGTTGG-3’	
IL-18BPd	P1-5’GACTGTTGCTTCCCAGAGGA-3’	NM_010531.1
	P2-5’AGGACCCACCAAGAACTGG-3’	
IL-18	P1-5’CAAACCTTCCAAATCACTTCCT-3’	NM_008360.1
	P2-5’TCCTTGAAGTTGACGCAAGA-3’	
ROR-α	P1-5’-TTACGTGTGAAGGCTGCAAG-3’	NM_013646.1
	P2-5’-GGAGTAGGTGGCATTGCTCT-3’	
MCP-1	P1-5’CCTTTCTTTCGTTTATGATTCACAT-3’	AJ238892
	P2-5’CCGCTGCAAGCTTCAGTTA-3’	
ROR-yt	P1-5’-CACTGCCAGCTGTGTGCT-3’	AF163668.1
	P2-5’TGCAAGGGATCACTTCAATTT-3’	
FOXP3	P1-5’- AGAAGCTGGGAGCTATGCAG-3’	BC132333.1
	P2-5’- GCTACGATGCAGCAAGAGC-3’	
RANTES	P1-5’- CTACTCCCACTCCGGTCCT-3’	M77747.1
	P2-5’-GATTTCTTGGGTTTCGTGGTC-3’	

BMP2	P1-5’CGGACTGCGGTCTCCTAA-3’	NM_007553.2
	P2-5’-GGGGAAGCAGCAACACTAGA-3’	
TNF-α	P1-5’-TCTTCTCATTCCTGCTTGTGG-3’	NM_013693.2
	P2-5’-GGTCTGGGCCATAGAACTGA-3’	
WNT10B	P1-5’TTCACGAGTGTCAGCACCA-3’	U61970.1
	P2-5’AAAGCACTCTCACGGAAACC-3’	
IL18BPa(Human)	P1-5’GGCGGGACAGAATTGATCT-3’	XM_0067184062
	P2-‘CTGGTGTCCAGTTGTGTCTCA-3’	
RUNX2	P1-5’CATGTTCAGCTTTGTGGACCT-3’	AF053956.1
	P2-5’GCAGCTGACTTCAGGGATGT-3’	
